# A Protochlorophyllide (Pchlide) *a* Oxygenase for Plant Viability

**DOI:** 10.3389/fpls.2019.00593

**Published:** 2019-05-15

**Authors:** Steffen Reinbothe, Sandra Bartsch, Claudia Rossig, Manli Yang Davis, Shu Yuan, Christiane Reinbothe, John Gray

**Affiliations:** ^1^Laboratoire de Génétique Moléculaire des Plantes and Biologie Environnementale et Systémique (BEeSy), Université Grenoble Alpes, Grenoble, France; ^2^OraSure Technologies Inc., Bethlehem, PA, United States; ^3^College of Resources, Sichuan Agricultural University, Chengdu, China; ^4^Department of Biological Sciences, The University of Toledo, Toledo, OH, United States

**Keywords:** chlorophyll biosynthesis, evolution of Rieske non-heme oxygenases, PTC52 structure-function relationships, chloroplast biogenesis, protein translocation

## Abstract

Higher plants contain a small, 5-member family of Rieske non-heme oxygenases that comprise the inner plastid envelope protein TIC55, phaeophorbide *a* oxygenasee (PAO), chlorophyllide *a* oxygenase (CAO), choline monooxygenase, and a 52 kDa protein (PTC52) associated with the precursor NADPH:protochlorophyllide (Pchlide) oxidoreductase A (pPORA) A translocon (PTC). Some of these chloroplast proteins have documented roles in chlorophyll biosynthesis (CAO) and degradation (PAO and TIC55), whereas the function of PTC52 remains unresolved. Biochemical evidence provided here identifies PTC52 as Pchlide *a* oxygenase of the inner plastid envelope linking Pchlide *b* synthesis to pPORA import. Protochlorophyllide *b* is the preferred substrate of PORA and its lack no longer allows pPORA import. The Pchlide *b*-dependent import pathway of pPORA thus operates in etiolated seedlings and is switched off during greening. Using dexamethasone-induced RNA interference (RNAi) we tested if PTC52 is involved in controlling both, pPORA import and Pchlide homeostasis *in planta*. As shown here, *RNAi* plants deprived of *PTC52* transcript and PTC52 protein were unable to import pPORA and died as a result of excess Pchlide *a* accumulation causing singlet oxygen formation during greening. In genetic studies, no homozygous *ptc52* knock-out mutants could be obtained presumably as a result of embryo lethality, suggesting a role for PTC52 in the initial greening of plant embryos. Phylogenetic studies identified PTC52-like genes amongst unicellular photosynthetic bacteria and higher plants, suggesting that the biochemical function associated with PTC52 may have an ancient evolutionary origin. PTC52 also harbors conserved motifs with bacterial oxygenases such as the terminal oxygenase component of 3-ketosteroid 9-alpha-hydroxylase (KshA) from *Rhodococcus rhodochrous*. 3D-modeling of PTC52 structure permitted the prediction of amino acid residues that contribute to the substrate specificity of this enzyme. *In vitro*-mutagenesis was used to test the predicted PTC52 model and provide insights into the reaction mechanism of this Rieske non-heme oxygenase.

## Introduction

The endosymbiotic theory explains the origin of mitochondria and plastids through the engulfment of bacteria into (proto) eukaryotic cells (Margulis, 1970). Primitive cyanobacteria are generally accepted to represent the ancestral prokaryotes that gave rise to chloroplasts (Gray, 1992; Archibald, 2012). It is estimated that an ancestral organelle, the “protoplastid,” may have arisen after ca. 90% of the total gene transfer from the genome of the cyanobacterial endosymbiont to the host cell nucleus had taken place and after an envelope protein import machinery had evolved that allowed the gene products to be imported back into the semiautonomous photosynthetic organelle (Martin and Müller, 1998; Martin et al., 1998). It was for a long time believed that most of the proteins destined to the primordial chloroplast acquired cleavable NH_2_-terminal transit sequences for import (Keegstra et al., 1989). However, proteomic studies have led to the result that this view is too simple.

Kleffmann et al. (2004) found that of the 604 chloroplast proteins identified in *Arabidopsis thaliana* only 376 contained predictable NH_2_-terminal transit sequences. Of the remainder, 37 were predicted to have a mitochondrial targeting signal, 40 to have a signal peptide for translocation into the endoplasmic reticulum, and 142 to possess no cleavable presequence. Evidence is emerging for the dual targeting of cytosolic proteins to mitochondria and chloroplasts (Peeters and Small, 2001), for the plastid import of transit peptide-less precursors (Miras et al., 2002, 2007; Nada and Soll, 2004; Rossig et al., 2013, 2014), and for the involvement of the secretory pathway in the import of certain precursors into chloroplasts (Villarejo et al., 2005; Baslam et al., 2016).

Chloroplast precursor proteins containing cleavable NH_2_-terminal transit sequences interact with translocon complexes of the outer and inner plastid envelope membranes, called the TOC and TIC machineries (Schnell et al., 1997; Inaba and Schnell, 2008). Pioneering work performed for pea chloroplasts identified the TOC complex to consist of three core components: TOC159, TOC75, and TOC34 (Hirsch et al., 1994; Kessler et al., 1994; Perry and Keegstra, 1994; Schnell et al., 1994; Tranel et al., 1995; Ma et al., 1996; Kouranov and Schnell, 1997; Bölter et al., 1998; Chen et al., 2000; Jelic et al., 2002). Likely in concert, these three proteins mediate the recognition, binding and translocation of the cytosolic precursors across the outer plastid envelope membrane (see Bedard and Jarvis, 2005; Hofmann and Theg, 2005; Kessler and Schnell, 2006; Shi and Theg, 2011; Paila et al., 2015; for reviews).

Biochemical and molecular genetic studies performed using *A. thaliana* have challenged the view that all of the different transit peptide-containing cytosolic precursors would enter the organelle through the same, TOC159/TOC75/TOC34 import complex. Bauer et al. (2000) identified two TOC proteins that complement the previously discovered main preprotein receptor protein TOC159. All three proteins share conserved GTP binding domains and membrane anchors but differ in the length of their NH_2_-terminal, cytosolically exposed domains implicated in precursor binding (Bauer et al., 2000; Ivanova et al., 2004). A fourth member of this GTP-binding receptor protein family, AtTOC90, was discovered later (Hiltbrunner et al., 2004). Pull-down and genetic assays confirmed that while AtTOC159 is involved in the import of photosynthesis-related precursor proteins, AtTOC120 and AtTOC130 are responsible for the import of other, non-photosynthetic proteins (Smith et al., 2004). Furthermore, it was shown that a TOC regulatory GTP-binding protein consists of twin components, termed AtTOC33 and AtTOC34, which exhibit different precursor specificities and expression patterns during plant development (Jarvis et al., 1998; Gutensohn et al., 2000; Jelic et al., 2003; Kubis et al., 2004). Last but not least, the ß-barrel protein TOC75, which establishes a hydrophilic translocation channel through which the majority of the cytosolic precursors are transported across the outer envelope (Hinnah et al., 1997, 2002), is encoded by three genes in *A. thaliana* of which two have different expression patterns and presumed functions (Baldwin et al., 2005). Increasing evidence supports the notion of multiple, regulated TOC complexes in the outer chloroplast envelope.

The TIC complex is less well characterized than the TOC complex. It consists of at least three core components: TIC110 (Kessler and Blobel, 1996; Lübeck et al., 1996; Inaba et al., 2003, 2005; Kovacheva et al., 2005; Balsera et al., 2008), TIC40 (Wu et al., 1994; Stahl et al., 1999; Chou et al., 2003; Kovacheva et al., 2005; Bédard et al., 2007; Chiu and Li, 2008) and a caseinolytic protein (Clp) C-class HSP93 chaperone (Akita et al., 1997; Nielsen et al., 1997; Constan et al., 2004; Kovacheva et al., 2005; Chou et al., 2006). Other, presumably auxiliary components were previously proposed to comprise TIC55 (Caliebe et al., 1997), TIC22 and TIC20 (Kouranov et al., 1998, 1999; Chen et al., 2002), TIC32 and TIC62 (Küchler et al., 2002; Balsera et al., 2010). TIC55 is an example of a protein previously thought to be associated with higher plant chloroplast protein import for which a cyanobacterial homolog was obtained (Caliebe et al., 1997. Phylogenetic analyses revealed that TIC55 belongs to a family of non-heme oxygenase proteins sharing conserved Rieske and mononuclear iron binding domains in plants and bacteria (Caliebe et al., 1997; Gray et al., 2004; Gross and Bhattacharya, 2009). Recent data identified a biochemical function for TIC55 as hydroxylase of phyllobilins, the products of chlorophyll breakdown, during plant senescence (Hauenstein et al., 2016), suggesting that TIC55 may exert multiple roles at different stages of plant development. A similar conclusion was drawn for TIC40 that is a multifunctional protein operating in protein import and thylakoid biogenesis (Bédard et al., 2017). Other members of the non-heme Rieske-mononuclear iron protein family, to which TIC55 belongs, include chlorophyll(ide) (Chl[ide]) *a* oxygenase (CAO) (Espineda et al., 1998; Tanaka et al., 1998), choline monooxygenase (CMO) (Rathinasabapathi et al., 1997), phaeophorbide *a* oxygenase (PAO) (Pruzinska et al., 2003), which is identical with the lethal leaf spot protein (LLS1) (Gray et al., 1997, 2002), and a 52 kDa protein associated with the precursor NADPH:protochlorophyllide (Pchlide) oxidoreductase (pPOR) A translocon of chloroplasts (PTC; Reinbothe et al., 2004a,b). PORA is a unique enzyme of the chlorophyll biosynthesis pathway that forms larger complexes with its twin component PORB and establishes unique light-harvesting complexes driving chlorophyll biosynthesis and, at the same time, conferring photoprotection onto etiolated seedlings during greening (Reinbothe et al., 1999; Reinbothe C. et al., 2003; Reinbothe S. et al., 2003; Buhr et al., 2008, 2017). PORA and PORB differ in their substrates specificities, with PORA being specific for Pchlide *b* and PORB being specific for Pchlide *a* (Reinbothe et al., 1999; Reinbothe S. et al., 2003). Both the pPORA and pPORB are encoded in nuclear DNA and are synthesized as larger precursors in the cytosol. Whereas the import of pPORA is substrate (Pchlide)-dependent, that of pPORB is not (Reinbothe et al., 1995a,b). Previous experiments suggested that PTC52’s function might be that of a Pchlide *a* oxygenase that ties Pchlide *b* synthesis from Pchlide *a* to pPORA import (Reinbothe et al., 2004a). Here, we examined the role of PTC52 using a combination of biochemical, cell biological and genetic approaches and provide evidence for an essential role of PTC52 for controlling Pchlide homoeostasis and pPORA import *in planta*.

## Results

### Identification of PTC52 as Protochlorophyllide *a* Oxygenase

PTC52 of barley was isolated by its co-purification with pPORA in import intermediates trapped in junction complexes between the outer and inner plastid envelope membranes at 0.1 mM Mg-ATP and 0.1 mM Mg-GTP (Reinbothe et al., 2004a). The determined partial amino acid sequence of barley PTC52 (HvPTC52) corresponds to EST clone BF266467. This EST is predicted to contain a Rieske (CxHx_16-17_Cx_2_H) and mononuclear iron binding (Nx_2_Dx_3-4_Hx_4_H) motif and the predicted amino acid sequence is most similar to the At4g25650 gene product of *A. thaliana* (AtPTC52).

A full-length cDNA was isolated corresponding to At4g25650. The predicted amino acid sequence (Supplementary Figure S1) identified the protein to consist of 536 amino acids, with a calculated *M_r_* of 61.3 kDa. Its basic pI of 8.9 was reminiscent of that for proteins of the inner plastid envelope membrane (Ferro et al., 2002, 2003). PTC52 is predicted to contain several *trans*-membrane segments and an amino terminal transit peptide for plastid import, beginning at position 55 (Supplementary Figure S1).

**FIGURE 1:**
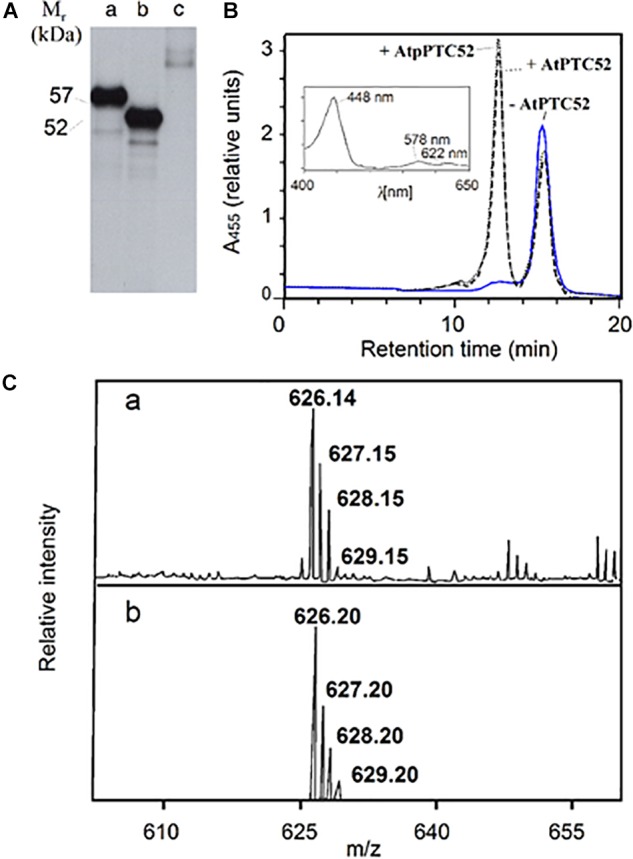
Activity of AtPTC52. **(A)** SDS-PAGE and autoradiography of ^35^S-AtpPTC52 (lane 1) and ^35^S-AtPTC52 (lane 2) translated in a wheat germ extract. Lane 3 shows the naked vector DNA control. **(B)** Pchlide *a* oxygenase activity of AtpPTC52 (dotted line) AtPTC52 (dashed line) and of the respective control without At(p)PTC52 (solid line). The curves show HPLC tracings of acetone-extracted pigments monitored at 455 nm by a photodiode array detector and the inset depicts an absorption spectrum of the peak eluting at 12.5 min. **(C)** Confirmation of the identity of Pchlide *b* eluting at 12.5 min by matrix-assisted laser desorption/ionization mass spectroscopy **(a)** and theoretical isotopic distribution of C35H30N4O6Mg corresponding to Pchlide *b* **(b)**. The matrix used was terthiophene (molecular weight 248.4).

cDNAs were generated encoding COOH-terminal hexa-histidine (His)_6_-tagged versions of PTC52 containing or lacking the predicted NH_2_-terminal chloroplast transit peptide (henceforth referred to as AtpPTC52 and AtPTC52, respectively). Upon *in vitro* transcription/translation, these constructs gave rise to ≈57 and ≈52 kDa bands, respectively (Figure 1A, lanes a and b), while naked vector DNA controls did not provide such specific bands (Figure 1A, lane c).

Activity measurements were carried out for AtpPTC52 as well as AtPTC52 using wheat germ-translated and bacterially expressed, purified protein. Incubations were conducted with Pchlide *a*, O_2_, ferredoxin (FD), and a FD-reducing system comprising glucose-6-phosphate, NADPH, glucose-6-phosphate dehydrogenase and FD:NADPH oxidoreductase (FNR). Figure 1B shows representative HPLC tracings of pigments formed in the absence of AtpPTC52 and AtPTC52 (solid line) or in the presence of AtpPTC52 and AtPTC52 (dotted and dashed lines, respectively). Pchlide *a* has a retention time of 15 min on the C18 HPLC column. The second peak, which was formed only in the presence of the AtpPTC52 and AtPTC52 proteins, eluted at 12.5 min. Absorbance measurements of this second peak using a photodiode array detector identified a major absorption maximum at 448 nm and two lower maxima at 578 and 622 nm, respectively (Figure 1B, inset). These maxima corresponded to values reported previously for Pchlide *b*: the so-called Soret band (448 nm), the *Q*_x_ band (578 nm) and the *Q*_y_ band (622 nm) (Scheumann et al., 1999; Oster et al., 2000; Reinbothe S. et al., 2003). The *Q*_x_ band had a higher absorbance than the *Q*_y_ band, which is typical for all proto *b* pigment species (Scheumann et al., 1999; Oster et al., 2000; Reinbothe S. et al., 2003). Matrix-assisted laser desorption/ionization spectroscopy proved the identity of PTC52-derived Pchlide *b*. As shown in Figure 1C, the major peak was at *m/z* 626.1 and had the same isotopic distribution as expected from the chemical formula of Pchlide *b*. When the HPLC tracings were compared, the measured low decrease in Pchlide *a* levels after incubation with the AtpPTC52 and AtPTC52 proteins correlated with an apparently large, fivefold increase in the amount of Pchlide *b* (Figure 1B, dotted and dashed lines), reflecting the fivefold difference in the absorption coefficients of Pchlide *a* and Pchlide *b* at the chosen wavelength of 455 nm (Scheumann et al., 1999; Reinbothe S. et al., 2003). Pchlide *a* to Pchlide *b* conversion required O_2_ and a FD-reducing system. In the absence of these factors, no Pchlide *b* was produced (data not shown). With wheat germ extract that had been programmed with the naked vector DNA and thus lacked the AtpPTC52 and AtPTC52 proteins, no Pchlide *b* was detectable (Figure 1B, solid line), indicating that Pchlide *b* formation is an enzymatic reaction requiring the At4g25650 gene product.

### Import of AtpPTC52 Into Isolated Chloroplasts

Given that AtPTC52 contains a predictable chloroplast transit peptide (Supplementary Figure S1), it was likely to enter the chloroplast through the general import pathway. Wheat germ-translated ^35^S-AtpPtc52 was therefore incubated with isolated, energy-depleted barley and Arabidopsis chloroplasts either in the absence of added nucleoside triphosphates (used to study energy-independent binding) or in the presence of (i) 0.1 mM Mg-ATP (used to study energy-dependent binding), (ii) 0.1 mM Mg-ATP plus 0.1 mM Mg-GTP (used to study the insertion of the precursor into the respective import apparatus) or (iii) 2 mM Mg-ATP plus 0.1 mM Mg-GTP (used to study import) (Kouranov and Schnell, 1997). After incubation, intact chloroplasts were re-isolated on Percoll.

**FIGURE 2:**
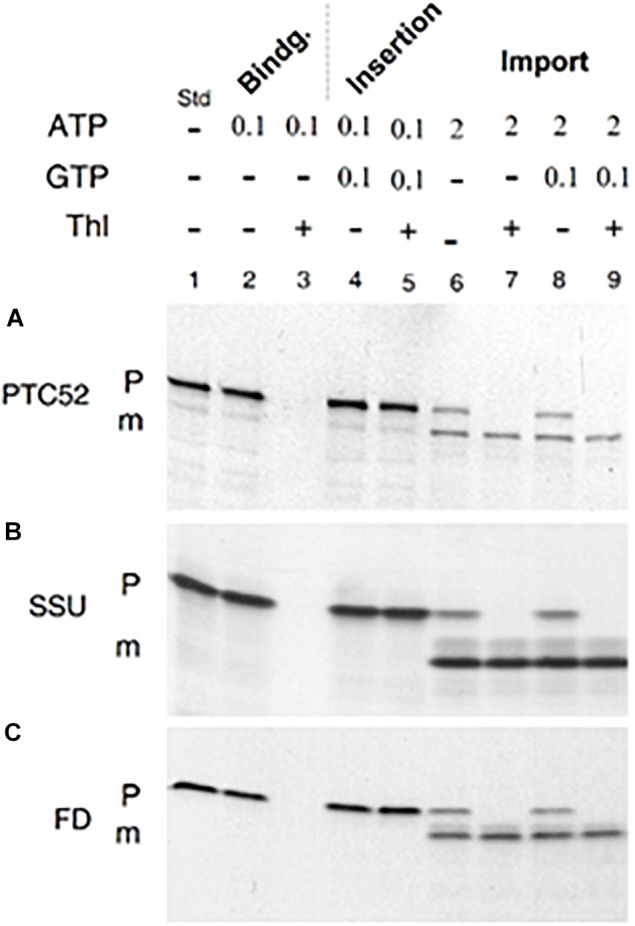
*In vitro*-import and processing of AtpPTC52 with isolated barley chloroplasts. **(A)** Plastid binding, insertion, and import of ^35^S-AtpPTC52. After incubation in the presence of the indicated concentrations of Mg-GTP and Mg-ATP (in mM), the plastids were resedimented, treated with or without thermolysin (Thl), and the amount of precursor and mature proteins bound to the plastids were determined by SDS-PAGE and autoradiography. **(B)** as in **(A)**, but showing plastid binding, insertion, and import of the small subunit precursor of ribulose-1,5-bisphosphate carboxylase/oxygenase (pSSU). **(C)** as in **(A)**, but depicting the binding, insertion, and import for the precursor of ferredoxin (pFD). P and m mark precursors and mature proteins, respectively; Std defines ^35^S-input standards.

In the absence of nucleoside triphosphates, faint amounts of pPTC52 were detected for re-isolated barley and Arabidopsis chloroplasts (data not shown). In the presence of 0.1 mM Mg-ATP, binding of ^35^S-AtpPTC52 to chloroplasts was greatly stimulated, allowing ca. 70–80% of precursor to be recovered in the plastid fraction obtained after re-centrifugation of the import assays (Figure 2A). Sensitivity toward thermolysin revealed that ^35^S-AtpPTC52 was bound to, but had not inserted into, the respective import machinery of the outer membrane of the chloroplast (Figure 2A, compare lanes 2 and 3). Thermolysin is a protease which degrades only surface-exposed precursors (Cline et al., 1984). When incubations were performed with 0.1 mM Mg-GTP and 0.1 mM Mg-ATP, ^35^S-AtpPTC52 attained a largely thermolysin-resistant conformation indicative of its insertion into the respective import machinery (Figure 2A, lanes 4 and 5). The use of sucrose instead of sorbitol in the plastid isolation and incubation buffers may explain this result and why at these nucleoside triphosphate concentrations other groups did not observe tight interactions of the precursors studied with the protein import machinery. Sucrose is known to confer a greater stability to membrane assemblies than sorbitol both *in vitro* and *in vivo* (Crowe and Crowe, 1984; Crowe et al., 1984) and thus could stimulate the early steps of import occurring at 0.1 mM Mg-GTP and 0.1 mM Mg-ATP. When the Mg-ATP concentration was raised to 2 mM, pPTC52 entered a productive import pathway and was processed to mature size (Figure 2A, lanes 8 and 9). Precursor translocation and processing did not require Mg-GTP (Figure 2A, lanes 6 and 7), as found in other studies (Inoue and Akita, 2008). Because the nucleoside triphosphate requirements of binding and import of pPTC52 were similar to those found for ^35^S-SSU and ^35^S-FD (Figure 2B,C), we concluded that pPTC52 entered the chloroplast through the general import pathway.

### AtPTC52 Forms Larger Chloroplast Envelope Membrane Complexes

**FIGURE 3:**
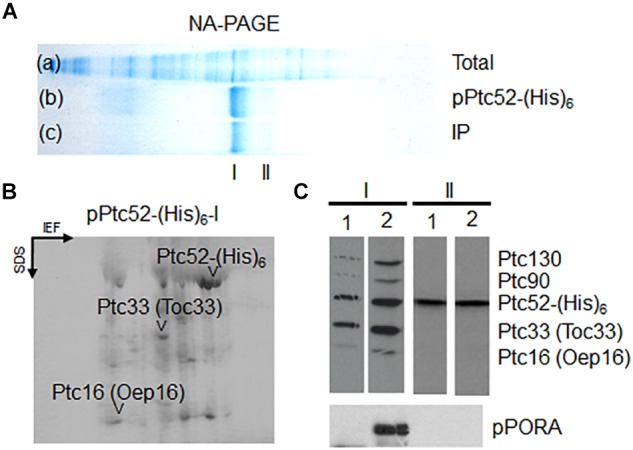
Production of higher molecular mass complexes containing AtpPTC52-(His)_6_ in barley chloroplasts. **(A)** Non-denaturing gel showing total envelope proteins and complexes prior to (lane a: *Total*) and after (lane b: *PTC52-(His)* an *in vitro*-import reaction with AtpPTC52-(His)_6_ as well as respective immunoprecipitates of purified AtpPTC52-(His)_6_ complexes (lane c: *IP*). Band I and band II designate PTC52-containing bands. **(B)** 2D-SDS-PAGE comprising isoelectric focusing (IEF) in the first dimension and SDS-PAGE in the second dimension of proteins bound to AtpPTC52-(His)_6_ in barley chloroplasts. The spot marked PTC52 is due to AtPTC52-(His)_6_ used as bait to isolate the complex, whereas other highlighted spots comprise PTC47, PTC33/TOC33, and PTC16/OEP16 and three unidentified spots of 20, 22, and 27 kDa (arrowheads)_._ **(C)** Detection of PTC proteins bound to AtpPTC52-(His)_6_ in barley chloroplasts and etiochloroplasts. AtpPTC52-(His)_6_-containing bands I and II were established with chloroplasts (lanes 1 and 3) and etiochloroplasts (lane 2 and 4), respectively. Protein bound to AtpPTC52-(His)_6_ then was purified from detergent-solubilized envelopes by Ni-NTA chromatography and subjected to 1D-SDS-PAGE. Proteins were detected by Western blotting using a mixed antiserum against the indicated PTC proteins (upper panels) or POR antiserum (lower panel).

^35^S-AtpPTC52-(His)_6_ was expressed in *Escherichia coli*, purified and incubated with isolated, energy-depleted barley chloroplasts in the presence of 0.1 mM Mg-GTP and 0.1 mM Mg-ATP. Then, mixed outer and inner envelope membranes were isolated from ruptured chloroplasts and solubilized with 1.3% decyl maltoside. Protein complexes containing AtpPTC52-(His)_6_ were purified by Ni-NTA chromatography and analyzed further by non-denaturing, analytical PAGE and Western blotting (Reinbothe et al., 1990). Pilot experiments revealed that AtPTC52-(His)_6_ was able to establish larger complexes after import (Supplementary Figure S2). When AtPTC52-(His)_6_-containing complexes were established with 2.5 nmoles of AtpPTC52-(His)_6_, one main band and a second, weaker band were obtained [Figure 3A, lane b, *Ptc52-(His)_6_*, cf. band I versus band II]. Both bands contained PTC52 and therefore could be detected with anti-PTC52 serum (Figure 3A, lane c, *IP*). Two-dimensional gel electrophoresis of the Ni-NTA-purified AtPTC52-(His)_6_ complex gave rise to a mixture of proteins comprising PTC47, PTC33, PTC/OEP16 and three other spots of approximately 27, 22, and 20 kDa (Figure 3B). Due to the presence of two distinct PTC52 populations comprising the endogenous barley PTC52 (HvPTC52) and added Arabidopsis PTC52 [AtPTC52-(His)_6_], however, most of these PTC proteins were present in sub-stoichiometric amounts as compared to the excess of added AtPTC52-(His)_6_ (Figure 3B,C). An explanation could be that PTC assembly occurs only during the actual translocation step of the pPORA when components of both the outer and inner plastid envelope closely interact. As shown previously, PTC formation is strongly favored in the presence of pPORA (Reinbothe et al., 2004a, 2005). In agreement with this view, the amounts of PTC130, PTC33, PTC16, and PTC proteins were drastically increased when AtPTC52-(His)_6_ was imported into etiochloroplasts from dark-grown plants that had been exposed to light for 2 h, accumulating large amounts of the endogenous pPORA *in vivo* (Reinbothe et al., 1996b). Consistent with previous results (Reinbothe et al., 1996b, 2000, 2006), significant amounts of the cytosolic pPORA then accumulated at the envelope of etiochloroplasts and established strong interactions with PTC52 and the other PTC proteins that allowed for their co-purification upon pPTC52-(His)_6_ import (Figure 3C, panel I, compare lane 2 versus lane 1). By contrast, band II seen in Figure 3A contains the unassembled exogenously added AtPTC52-(His)_6_ as well as endogenous barley PTC52 presumably competed out from preexisting envelope complexes by the added bait protein (cf. Supplementary Figure S3).

### Inhibition of pPORA Import and PTC52 Activity by Diethyl Pyrocarbonate

**FIGURE 4:**
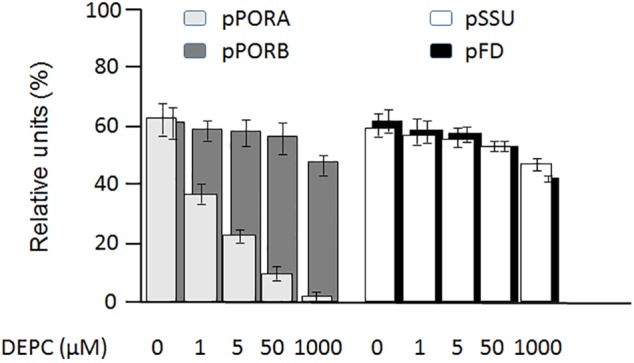
Differential inhibition by diethyl pyrocarbonate (DEPC) of ^35^S-PORA (light gray columns), ^35^S-PORB (dark gray columns), ^35^S-SSU (white columns) and ^35^S-FD (black columns) import into isolated Arabidopsis chloroplasts. Incubations were carried out in the presence of 0 μM (lane a), 1 μM (lane b), 5 μM (lane c), 50 μM (lane d), and 1 mM DEPC (lane e). Percentages refer to the amount of thermolysin-protected mature proteins in re-isolated chloroplasts relative to the amount of input radioactivity, set as 100. Error bars refer to four independent experiments.

PTC52 contains highly conserved His residues in its Rieske iron-sulfur cluster (Supplementary Figure S1) and was therefore expected to be sensitive to ethoxyformylating compounds such as diethyl pyrocarbonate (DEPC) (Ohnishi et al., 1994). To test the involvement of His residues in the PTC52 reaction, plastid import reactions were conducted for ^35^S-pPORA, ^35^S-pSSU, ^35^S-pPORB and ^35^S-pFD in the presence and absence of DEPC. To permit the Pchlide-dependent import of pPORA, isolated plastids were pretreated with the Pchlide precursor 5-aminolevulinic acid and re-purified (Reinbothe et al., 1995a). In four replicate samples, dose-response relationships were established for 1, 5, 50, or 1000 μM concentrations of DEPC. Figure 4 shows that only import of ^35^S-pPORA, but not that of the other tested precursors, was inhibited at low, 1, 5, and 50 μM concentrations of DEPC. In the presence of 1 mM DEPC, no ^35^S-pPORA import occurred, while import of ^35^S-pSSU, ^35^S-pPORB and ^35^S-pFD was reduced by only 12, 14, and 8%, respectively, of control levels measured in mock assays lacking DEPC (Figure 4).

To further explore the roles of PTC52 as DEPC target, activity measurements were carried out using the *in vitro*-expressed and purified COOH-terminally His-tagged AtPTC52 and HvPTC52. For comparison, PTC-bound AtPTC52 and HvPTC52 proteins were isolated and used in the enzyme assay. Table 1A shows that wheat germ-translated AtPTC52 and HvPTC52 were likewise sensitive to DEPC *in vitro*. In either case, the inhibitor dropped the activity to almost undetectable levels. Similar results were obtained for PTC complexes containing AtPTC52 and HvPTC52 (Table 1B). This result proved the involvement of His residues in the catalytic mechanism of PTC52.

**Table 1:** Pchlide *a*-oxygenase activity of *in vitro*-expressed or PTC-bound PTC52 in barley and *A. thaliana* chloroplasts.

Protochlorophyllide a-Oxygenase Activity (nkat⋅mg^-1^ PTC52 protein)				
	**Barley**		**Arabidopsis**	
	**- DEPC**	**+ DEPC**	**- DEPC**	**+ DEPC**
**(A)**
+ Supplements	3.8 ± 0.20	0.10 ± 0.01	4.0 ± 0.2	0.14 ± 0.02
- Supplements	n.d.	n.d.	n.d.	n.d.
**(B)**
+ Supplements	4.2 ± 0.15	0.14 ± 0.01	4.8 ± 0.25	0.16 ± 0.02
- Supplements	0.42 ± 0.35	n.d.	0.48 ± 0.05	n.d.

*Wheat germ-translated PTC52 **(A)** or PTC complex-bound PTC52 **(B)** from barley or A. thaliana was used for the activity measurements. The assays contained glucose-6-phosphate, NADPH, glucose-6-phosphate dehydrogenase and differed by the presence (+ supplements) or absence (- supplements) of FD and FD:NADPH oxidoreductase (FNR). Pchlide a to Pchlide b conversion was analyzed by HPLC as described in Figure 1. Mean values refer to three independent experiments each.*

The data summarized in Table 1B additionally revealed that conversion of Pchlide *a* to Pchlide *b* by the isolated PTC complexes required the same FD and the FD-reducing system as the *in vitro*-reaction carried out by the soluble AtPTC52 and HvPTC52 proteins. However, low Pchlide *a* oxygenation was also detectable in the absence of these additives (Table 1B), suggesting that some of the PTC proteins may correspond to FD and FNR. Unlike Pchlide *a*, neither Chlide *a* nor phaeophorbide *a* was accepted as substrates in all of our tests (data not shown), confirming the previously determined stringent substrate specificity of HvPTC52 and AtPTC52 (Bartsch et al., 2008; Bartsch, 2009; Hauenstein et al., 2016).

### Dexamethasone-Induced RNA Interference (*RNAi*) to Examine the Role of PTC52 *in planta*

**FIGURE 5:**
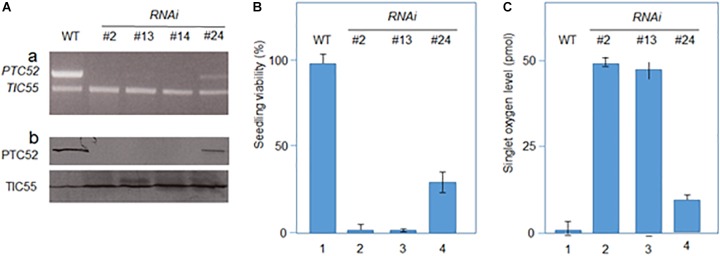
Characterization of RNAi seedlings. **(A)** Absence of *PTC52* transcript **(a)** and PTC52 protein **(b)** in independent lines after dexamethasone-(DEX)-induced RNAi, as revealed by RT-PCR. For comparison, *TIC55* transcript **(a)** and TIC55 protein **(b)** levels are indicated for RNAi versus wild-type (WT) seedlings. Note that residual *PTC52* transcript is present in RNAi line #24. **(B)** Viability of DEX-induced RNAi versus WT seedling, as assessed by tetrazolium staining. Percentages refer to means of three independent experiments. **(C)** Singlet oxygen production in DEX-induced 4.5 days-old etiolated RNAi versus WT seedlings after a 30-min white light exposure. Singlet oxygen production was measured using the DanePy reagent. Singlet oxygen levels are mean values derived from three independent experiments.

The results presented thus far implied that PTC52 operated as Pchlide *a* oxygenase that ties Pchlide *b* synthesis to pPORA import. Dexamethasone-induced RNA interference (*RNAi*) was used to test this hypothesis. Four days-old, etiolated Arabidopsis seedlings were deprived of *PTC52* transcript and PTC52 protein by a 12 h-DEX pre-treatment (Figure 5A; see also Supplementary Figures S4, S5) and their plastids used for protein uptake and pigment extraction experiments. Confirming our hypothesis, DEX-treated *RNAi* plants produced drastically reduced amounts of Pchlide *b* (Table 2) and were unable to import pPORA (Supplementary Figure S5). Tests with tetrazolium (Nortin, 1966) demonstrated a significant, light-induced reduction in seedling viability for DEX-treated plants, as compared to mock-treated plants (Figure 5B). Pigment analyses by HPLC demonstrated a ≈5-fold increase in the amount of Pchlide *a* in DEX-treated versus wild-type and mock-treated *RNAi* plants (Table 2). We tentatively concluded that excess Pchlide *a* accumulating in *RNAi* seedlings most likely operated as photosensitizer and triggered the production of singlet oxygen. As shown previously, singlet oxygen is a powerful cytotoxin but cell death signaling compound (Reinbothe et al., 2010b). To test this idea, singlet oxygen measurements were conducted with DanePy, a dansyl-based compound that undergoes quenching of its fluorescence upon reacting with singlet oxygen (Hideg et al., 1998; Kálai et al., 2002). Figure 5C shows quantitative data for 4.5 days-old etiolated plants that had been infiltrated with DanePy and subsequently exposed to white light of 125 μE m^-2^ sec^-1^ for 30 min. While fluorescence quenching and thus singlet oxygen production was negligibly low for wild-type seedlings, that of *RNAi* seedlings was significantly enhanced.

**Table 2:** Pigment accumulation in *RNAi* plants lacking PTC52.

Pigment (pmol per g fresh weight)				
	**Wild-type**	**RNAi^(1)^**		
		**#1**	**#2**	**#3**
**Darkness (4.5 days-old)**				
Pchlide *a*	248 ± 22	1250 ± 60	1340 ± 82	1280 ± 44
Pchlide *b*	1060 ± 115	35 ± 4	38 ± 8	37 ± 5
**Illuminated (4.5 days-old + 4 h L)**				
Pchlide *a*	200 ± 18	20 ± 30		
Pchlide *b*	1030 ± 75	n.d.	n.d.	10 ± 3
Chlide *a*	55 ± 6	n.d.	n.d.	5 ± 1
Chlide *b*	26 ± 5	n.d.	n.d.	n.d.

*Seedlings were grown for 4 days in darkness and then sprayed with dexamethasone or mock-incubated for 12 h. Pigments were extracted with 100% acetone containing 0.1% diethylpyro carbonate (DEPC) and subjected to HPLC (Reinbothe C. et al., 2003; Reinbothe S. et al., 2003). In a parallel experiment, seedlings were treated identically except for exposing them to white light of 125 μE m^-2^ sec^-1^ for 4 h prior to harvest. Identification and quantification of HPLC-resolved pigments was made by absorbance measurements using a photodiode array detector and known standards. Mean values refer to three independent experiments. n.d. defines undetectable pigment levels. ^(1)^Pigment levels in mock-incubated RNAi seedlings are indistinguishable from those in the wild-type and are not shown here.*

### Identification of Conserved Functional Domains in PTC52 Proteins

The advent of complete genomes of many plant and algal species permitted the re-evaluation of the coding regions of PTC52-related proteins in order to identify common and unique domains of PTC52 with regard to PAO, TIC55, and CAO and illuminate the issue of substrate specificity. The full length proteins of PTC52, PAO, TIC55, and CAO proteins from two dicots (*A. thaliana* and *Populus trichocarpa*), two monocots (*Oryza sativa* and *Zea mays*) and the moss *Physcomitrella patens* were identified and aligned using CLUSTAl W (Supplementary Figure S6). In addition, CAO from *Chlamydomonas reinhardtii* and four related oxygenases from photosynthetic bacteria were included. Close inspection of this multiple sequence alignment revealed the presence of conserved Rieske centers, mononuclear Fe binding sites, and thioredoxin (TRX) target motifs (Supplementary Figure S6). In addition, motifs within variable regions of these proteins were identified that are likely to confer substrate specificity to the different family members (Supplementary Figure S6 and Supplementary Table S1).

**FIGURE 6:**
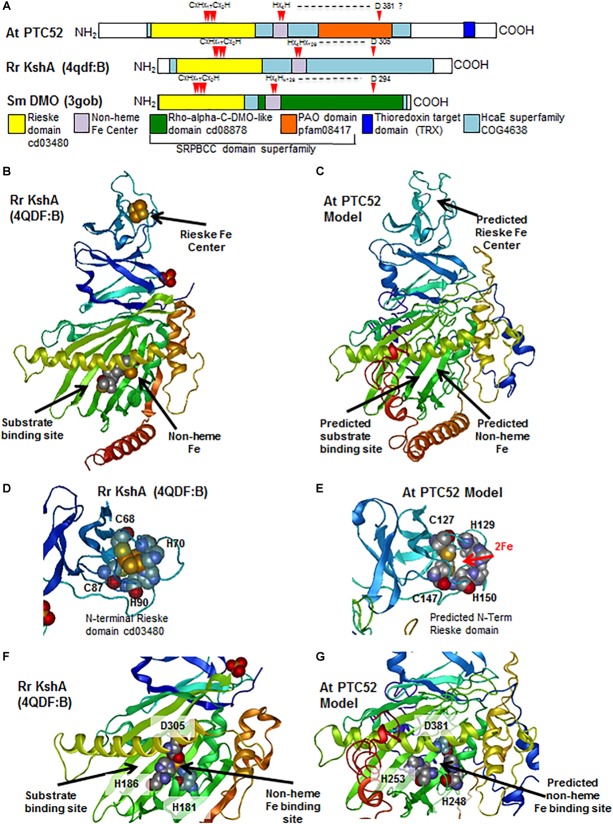
Characterization of conserved functional domains in PTC52 proteins by homology modeling. **(A)** Schematics of At PTC52, RrKshA, and SmDMO oxygenase proteins showing the locations of conserved functional domains and Fe binding residues. **(B)** Tertiary crystal structure of RrKshA (4QDF:B) from Penfield et al. (2014) displaying the Rieske, non-heme Fe binding, and steroid substrate binding/catalytic sites. Protein ribbon representation is colored by position from N-terminus (blue) to C–terminus (red). **(C)** Homology model of At PTC52 protein using the i-TASSER algorithm and displaying the predicted locations of Rieske, Non-heme Fe binding, and steroid substrate binding/catalytic sites. **(D)** Tertiary crystal structure of RrKshA (4QDF:B) Rieske domain with space filling representation of residues that coordinate iron binding. **(E)** Homology model of At PTC52 displaying the predicted locations of the residues within the Rieske domain that would bind Fe. **(F)** Tertiary crystal structure of RrKshA (4QDF:B) central domain with space filling representation of residues that coordinate non-heme iron binding and location of bound steroid substrate. **(G)** Homology model of At PTC52 displaying the predicted central domain and predicted residues coordinating non-heme Fe binding.

To further explore the possible structural conservation of these motifs within PTC52, the AtPTC52 protein was modeled against homologous proteins in the protein database^1^ using i-TASSER algorithm (Yang et al., 2015). Accordingly, PTC52 is most closely threaded with bacterial oxygenases such as the terminal oxygenase component of 3-ketosteroid 9-alpha-hydroxylase (KshA) from *R. rhodochrous* (TM-score 0.651) (Penfield et al., 2014) and Dicamba (2-methoxy-3,6-dichlorobenzoic acid) *O*-demethylase (DMO) from *Stenotrophomonas maltophilia* (TM-score 0.527)( (D’Ordine et al., 2009). KshA and DMO belong to the HcaE (COG4638) superfamily of proteins that include ring-hydroxylating dioxygenases, which like PTC52, harbor Rieske and mononuclear Fe binding centers that facilitate their enzymatic reactions (Figure 6A). The top ranked model (*C*-score -2.56) generated by threading AtPTC52 against KshA predicts an NH_2_-terminal location for the Rieske domain and an internal location for the non-heme Fe binding domain (Figure 6A,B). Closer examination of the model revealed that the NH_2_-terminal Rieske domain is distant from the catalytic site and likely surface-exposed (Figure 6C,D) which would facilitate the transfer of electrons to the non-heme site in a second subunit of a multimeric enzyme complex, as shown for KshA and DMO (D’Ordine et al., 2009; Penfield et al., 2014). The predicted non-heme binding site in PTC52 is found immediately adjacent to the presumed substrate binding site, as found for KshA where the reducing equivalents can be passed directly to the substrate, suggesting that the reaction mechanisms of PTC52 and KshA are similar (Figure 6F,G). The coordination of the non-heme Fe atom is facilitated by H181 and H186 and a distally located D305 on an α-helix that borders the substrate binding site (Figure 6E). Interestingly, the D381 residue of PTC52 is invariant amongst PTC52, PAO, CAO, and TIC55 proteins (Supplementary Figure S6) and homology modeling predicts that it is similarly located to co-ordinate non-heme Fe binding (Figure 6G).

In addition to the Rieske and non-heme iron binding domains previously identified within PTC52 and related PAO oxygenases, there is also a conserved central region (PAO domain cd03480) in this gene family (Figure 6A). This PAO domain belongs to the larger START/RHO_alpha_C/PITP/Bet_v1/CoxG/CalC (SRPBCC) ligand-binding domain superfamily (Acc. No. cl14643). SRPBCC domains are found in a variety of oxygenase enzymes that have a deep hydrophobic ligand-binding pocket that binds diverse substrates. The protein structure of several such oxygenases has been determined and suggests an overall structural conservation of this ligand binding pocket.

Three other motifs nearer the COOH terminus and in the vicinity of the thioredoxin target motif were found to be signature motifs for the PTC52 clade in land plants (motif E: LPPxP; motif F: AxKx_3_ALEx_2_LQx_4_Ax_2_Gx_2_A; motif G: AVx_5_SxWLx_2_Fx_2_K) (Supplementary Figure S6). Their predicted locations within the COOH-terminal α-helical regions suggest that they may play a role in subunit interactions or with other components of the complex identified in this study.

**FIGURE 7:**
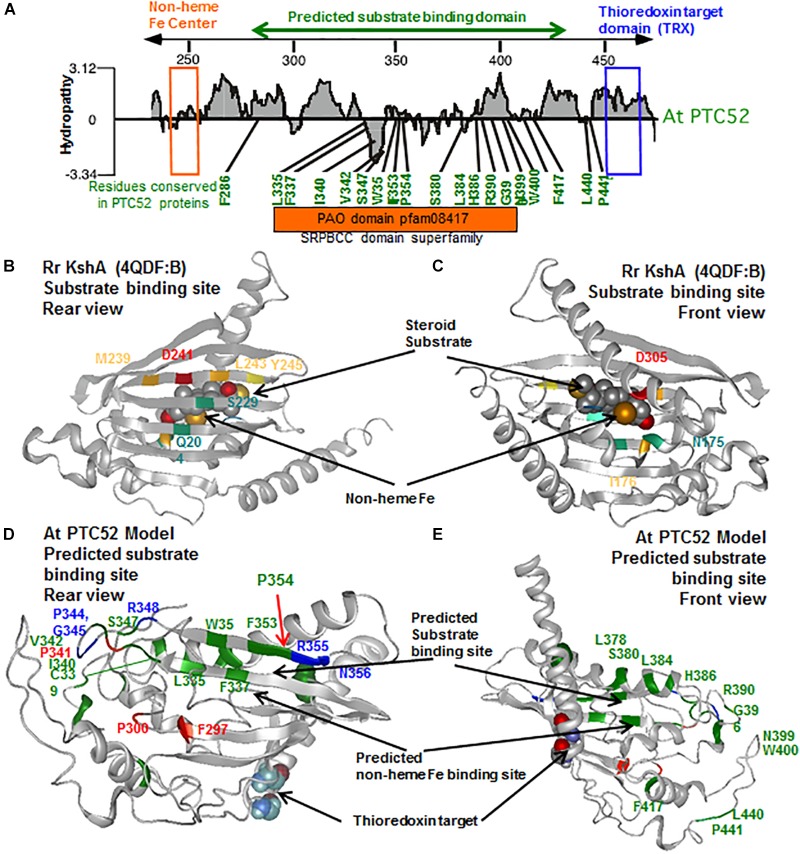
Identification of uniquely conserved residues in the predicted substrate binding site of PTC52 proteins. **(A)** Hydropathy plot of substrate binding region of PTC52 protein from *A. thaliana* (see also Supplementary Figure S7). The mononuclear iron binding motif is highlighted by an orange box and the thioredoxin target site is highlighted by a blue box. Residues that are uniquely conserved in the predicted substrate binding region amongst PTC52 proteins but not in PAO, TIC55 or CAO proteins are shown in green. See Supplementary Table S1 for further listing of conserved residues in this region amongst PTC52 related proteins. **(B,C)** Rear and front views of the tertiary crystal structure of Rr KshA (4QDF:B) from Penfield et al. (2014) displaying the central substrate binding domain with residues that coordinate substrate binding highlighted in color. **(D,E)** Rear and front views of homology model of At PTC52 displaying the predicted substrate binding site. Residues uniquely conserved amongst PTC52 proteins are displayed in green. Residues uniquely conserved amongst PTC52 and PAO proteins are displayed in blue. Residues invariant amongst PTC52, PAO, TIC55, and CAO proteins are displayed in red. C460 and C463 residues belonging to the thioredoxin target domain are shown as space filling molecules.

Lastly, the presence of the thioredoxin target region at the base of a COOH-terminal α-helix (Figure 7D) which may occlude the substrate binding site suggests a mechanism by which substrate accessibility or subunit interactions are influenced by cellular redox conditions as we have previously proposed (Reinbothe et al., 2006; Bartsch et al., 2008; Bartsch, 2009).

### Site-Directed Mutagenesis of PTC52

Site-directed mutagenesis was used to tentatively probe the robustness of the predicted 3D-model of PTC52. Taking into account the data summarized in Supplementary Figures S6, S7, as well as Supplementary Table S1) on the presence of unique amino acid residues distinguishing PTC52 from the closely related PAO family members, mutations were introduced replacing F286 (lying in a region preceding motif A of the predicted substrate binding pocket), F337, W351, P354 (all being part of motif B of the predicted substrate binding pocket), H386, R390, N399, W400 (all lying in the region linking motifs C and D of the predicted substrate binding pocket), F417, L440, P441, and P442 (all found in motif E of the predicted substrate binding pocket) by alanine (Ala, A) residues. In addition, deletions were made eliminating the mononuclear Fe binding E/D^241^N^242^x_2_Dx_2_Hx_4_H^253^ domain and F^297^xA/PPC/V^301^ and C/N^339^xP^343^ substrate binding signature motifs (Supplementary Table S1). Moreover, single and double mutants were created in which A247 and V249 in the mononuclear Fe binding E/D^241^N^242^x_2_Dx_2_Hx_4_H^253^ motif (the exact sequence is: E/D^241^N^242^LMD^245^PA^247^HV^249^PYAH^253^) were replaced by aspartate (Asp, D) residues. In addition, H248 and H253 that are shared between PTC52, PAO, TIC55, and CAO (Supplementary Table S1) were replaced by Ala residues. In a last set of experiments, we engineered PTC52 mutant proteins lacking the predicted Rieske (C^127^x_2_H^129^x_17_C^147^x_2_H^150^) domain or containing Ala substitutions instead of C127, H129, C147, or H150, respectively.

Activity measurements carried out in the presence of Pchlide *a*, FD, FNR and the other previously determined supplements (cf. Figure 5 and Table 1) showed that deleting the Rieske domain from PTC52 abolished Pchlide *a*-to-*b* conversion (Table 3). Replacing C127, H129, C147, or H150 residues by Ala residues led to ≈22–26% reductions of PTC52 activity. All four amino acids are evolutionarily conserved in PTC52, PAO, TIC55, and CAO (Supplementary Table S1) and are predicted to be located at the protein surface in our PTC52 model, suggesting that they participate in the transfer of electrons from FD/FNR to PTC52 and that this process was impeded in the engineered PTC52 mutant protein.

**Table 3:** Pchlide *a*-oxygenase activity of *in vitro*-expressed PTC52 mutant proteins.

Protochlorophyllide *a*-Oxygenase Activity (nkat⋅mg^-1^ PTC52 protein)		
**Point mutants**		
Rieske motif		
C127A	3.12	(22%)
H129A	2.98	(25.5%)
C147A	3.18	(36.4%)
H150A	3.02	(24.5%)
Mononuclear iron binding motif (241E/D242Nx2Dx2Hx4H253)		
A247D	3.00	(25.0%)
V249A	3.72	(7.0%)
A247D + V249A	2.75	(31.25ù)
H248A	2.82	(29.5%)
H253A	2.34	(41.5%
H248A + H253A	0.48	(88%)
Predicted substrate binding pocket		
Region adjacent to motif A		
F286A	3.98	(0.5%)
Motif B		
F337A	3.20	(20%)
W351A	3.38	(15.5%)
P354A	0.22	(94.5%)
Linker region between motives C and D		
H386A	4.00	(0%)
R390A	3.98	(0.5%)
N399A	4.00	(0%)
W400A	3.97	(0.75%)
Motif E		
F417A	4.00	(0%)
L440A	4.00	(0%)
P441A	3.24	(19%)
**Deletion derivatives**		
Rieske motif (AC127xxH12 9xl7C147x2H150)	n.d.	
Mononuclear iron binding motif (A[241E/D242Nx2Dx2Hx4H253])	n.d.	
Predicted substrate binding pocket		
Motif A		
(A[2 97FxA/P,P,C/V301])	3.92	(2%)
No amino acid residues unique to PTC52 found but at least two residues shared with PAO, that is, F297 and P400		
Motif B		
(A[339C/N, xP343])	3.08	(23%)
Only C339 is unique, whereas P343 is shared with PAO		

*PTC52-(His)_6_ mutant proteins were generated by site-directed mutagenesis, expressed in E. coli and purified from bacterial extracts by affinity chromatography. Activity tests in turn were carried out as described in Figure 1, using Pchlide a as substrate and glucose-6-phosphate, NADPH, glucose-6-phosphate dehydrogenase, FD and FD:NADPH oxidoreductase (FNR) as supplements (cf. Figure 5 and Table 1). PTC52 activities are mean values and refer to three independent experiments. Numbers in brackets define the percentage of drop in PTC52 activity in the generated mutant proteins relative to the wild-type PTC52 activity, set as 100%.*

Deleting the mononuclear Fe binding E/D^241^N^242^x_2_Dx_2_Hx_4_ H^253^ motif from PTC52 abrogated Pchlide *a*-to-*b* conversion and thus PTC52 activity (Table 3). In the A247D and V249A single and double mutants, PTC52 activity was reduced by ≈25 and 7%, respectively, in case of the single mutants, and by ≈31% in case of the A247D-V249A double mutant, relative to the wild-type PTC52 protein. Replacing H248 and H253 provided additive effects and dropped PTC52 activity by a total of ≈88%. These results indicated that the correct architecture of the E/D^241^N^242^x_2_Dx_2_H^248^x_4_H^253^ motif and correct positioning of hydrophobic amino acids (A247 and V249*)* as well as His residues (H248 and H253) play critical roles for catalysis. Introducing charged amino acid residues such as Asp presumably interferes with some hydrophobic interactions normally taking place between the enzyme and its substrate. On the other hand, H248 and H253 obviously accomplish key functions for fixing Fe in the mononuclear Fe binding E/D^241^N^242^x_2_Dx_2_H^248^x_4_H^253^ motif.

Deleting the F^297^xA/PPC/V^301^ motif (referred to as motif A in Supplementary Figure S7) from PTC52 only slightly reduced PTC52 activity (by ≈2% as compared to the wild-type activity) (Table 3). By contrast, deleting the C/N^339^xP^343^ motif (being part of motif B in Supplementary Figure S7) diminished PTC52-catalyzed Pchlide *a*-to-Pchlide *b* conversion by ≈23%, as compared to the wild-type activity (Table 3).

Single replacements of most of the identified fingerprint amino acid residues distinguishing PTC52 from PAO (Supplementary Table S1) had only little, if any, effects on PTC52’s catalytic activity (Table 3). Exceptions were PTC52 proteins containing single Ala residues in place of F337, W351, and P441, being present in motifs B (F337 and W351) and E (P441), respectively, of the predicted substrate binding pocket. For the D337A and W351A mutants, PTC52 activity was reduced by ≈18–22%, as compared to the wild-type activity (Table 3). The most severe effect was seen for the P354 substitution that gave rise to a largely inactive PTC52 protein (≈5–6% of wild-type activity; cf. Table 3). Interestingly, P354 flanks two conserved regions in motif B of the predicted substrate binding domain of PTC52 (Supplementary Figure S6) for which mutations in *acd1* (which binds the tetrapyrrole phaeophorbide *a*) provoke strong phenotypes (Greenberg and Ausubel, 1993; Gray et al., 1997; Pruzinska et al., 2003). Thus, P354 is likely to play a crucial role in the establishment of the substrate binding pocket of PTC52. By contrast, no comparable effects were seen for PTC52 mutant proteins with alterations in the fingerprint amino acid residues constituting the linker region between motifs C and D (Table 3). Similarly, single substitutions in presumed key amino acids in motif E did not affect PTC52 activity, except for replacements of P441 that gave rise to PTC52 protein with lowered activity (≈81% of wild-type activity; Table 3). Although our mutagenesis study is far from being comprehensive, our data validate our 3D-model and provide a basis for further in-depth analyses.

## Discussion

### PTC52 Is Involved in Pchlide *b* Synthesis and pPORA Import

NADPH:protochlorophyllide (Pchlide) oxidoreductases (POR) A and B are key enzymes of chlorophyll biosynthesis in angiosperms. As nuclear gene products, the PORA and PORB are synthesized as larger precursors in the cytosol and must be imported posttranslationally into etioplasts where they assemble into larger complexes conferring photoprotection during greening (see Reinbothe et al., 1996a,c, 2010a, for review). Plastid envelope proteins have been identified that interact with the cytosolic precursors of the PORA and PORB (pPORA and pPORB) during their posttranslational plastid import (Reinbothe et al., 2004a,b, 2005, 2015; Pollmann et al., 2007; Samol et al., 2011a,b). pPORA interacts with several previously unreported components including the presequence receptor protein TOC130, GTP regulatory subunit TOC33, outer plastid envelope protein OEP16, chaperone-like protein CDF1 (cell growth defective factor 1), and PTC52. The unique composition and function of the plastid envelope protein complex through which the pPORA is imported into etioplasts assures that Pchlide *b* synthesis is tied to pPORA translocation. pPORA is abundantly expressed in dark-grown seedlings but its level rapidly declines because of the combined negative effect of light on *PORA* gene transcription and mRNA stability (Armstrong et al., 1995; Holtorf et al., 1995; see Reinbothe et al., 1996c, 2010a, for review). Superimposed is a negative light effect on the level of Pchlide that is needed for the substrate-dependent import of the pPORA into the plastid compartment (Reinbothe et al., 1995a,b). Mature PORA thus is present in etioplasts, but its precursor, pPORA, accumulates in envelope import intermediates in etioplasts that undergo differentiation into chloroplasts (Reinbothe et al., 1996b). When chase experiments were performed in which the envelope-bound pPORA was allowed to translocate into the stroma in the presence of 0.5 mM 5-aminolevulinic acid plus 2 mM Mg-ATP and 0.1 mM Mg-GTP, increasing amounts of Pchlide *b* were synthesized and co-purified with the recovered import complex, as compared with the import complex obtained at time zero (Reinbothe et al., 2004a). These pioneering results suggested the possibility that PTC52 functioned as Pchlide *a* oxygenase.

In the present work, we examined the role of PTC52 *in vitro* and *in planta*. A cDNA was isolated for PTC52 of *A. thaliana* (AtPTC52) which confirmed that the protein contained the same Rieske and mononuclear iron binding motifs as barley PTC52 (Supplementary Figure S1). In addition, AtPTC52 contains the COOH-terminal CxxC thioredoxin motif found in other PTC52, TIC55, and PAO sequences (Supplementary Figures S6, S7). The mononuclear binding site may complex iron via two His residues and one carboxylate within the (ENx_2_Dx_2_Hx_4_H) motif forming a His-1-carboxylate facial triad (Lange and Que, 1998). As previously proposed (Bartsch et al., 2008), the CxxC thioredoxin motif may operate in dark/light regulation and/or oxidative control.

AtPTC52 is synthesized as a larger, ≈57 kDa precursor which is processed to mature size upon import into chloroplasts. Proteomic studies identified PTC52 as inner plastid envelope protein (Ferro et al., 2002, 2003; Bräutigam and Weber, 2009; Huang et al., 2013). Transient expression of green fluorescence protein (GFP)-tagged fusion protein in mesophyll protoplasts from senescent Arabidopsis wild-type leaves identified PTC52 in plastids (Hauenstein et al., 2016). As shown here, PTC52 is embedded into its target membrane presumably via up to four *trans*-membrane segments and interacts with TOC130, TOC33, and OEP16 to establish functional envelope complexes operative in Pchlide *a* oxygenation and pPORA import (Figure 2, 3; see also Supplementary Figures S2, S3).

Activity measurements demonstrated that AtPTC52 and HvPTC52 exhibit Pchlide *a*-oxygenase activity *in vitro* (Figure 1 and Table 1). The reaction catalyzed by AtPTC52 and HvPTC52 (Reinbothe et al., 2006) required Pchlide *a* and O_2_ as well as FD and a FD-reducing system, comprising FNR, glucose-6-phosphate and glucose-6-phosphate dehydrogenase. The same co-factor requirements were found for PTC complexes containing PTC52 from barley and *A. thaliana* chloroplasts (Table 1). Most likely, FD and FNR supplied electrons for the reaction. Jäger-Vottero et al. (1997) reported on the presence of electron transport chains comprising Fe-S clusters and other iron-sulfur proteins in envelope membranes of spinach chloroplasts that may involve PTC52, while not precluding the existence of other reaction chains. The fact that PTC52-related bacterial Rieske non-heme oxygenases, such as KshA, occur in two-component complexes with ferredoxin reductases (i.e., KshB) (Morawski et al., 2000; Petrusma et al., 2014) is suggestive of the presence of similar reductase complexes in the PTC52 reaction. The reaction catalyzed by PTC52 is an essential step in the Pchlide-dependent plastid import pathway of pPORA. Dys-regulation of PTC52’s function, as encountered in the generated *RNAi* plants, led to devastating greening defects and seedling lethality that may be explained by the failure to drain electrons from FD and transfer them to other cellular targets or overproduction of Pchlide *a* and subsequent pigment-sensitized singlet oxygen formation triggering cytotoxic effects and cell death signaling cascades (see below).

### Substrate Specificity of PTC52 and Related Proteins

The Arabidopsis genome encodes five Rieske-type oxygenases (Gray et al., 2002). Biochemical and genetic studies showed that four out of these five oxygenases are operative in chlorophyll metabolism. Two of them are involved in chlorophyll biosynthesis, i.e., CAO that catalyzes the oxidation of chlorophyll(ide) *a* to chlorophyll(ide) *b* (Tanaka et al., 1998; Philippar et al., 2007), and PTC52 that catalyzes the same type of reaction, but using Pchlide *a* as substrate. By contrast, PAO and TIC55 are operative during chlorophyll breakdown (Pruzinska et al., 2003). Whereas PAO acts as phaeophorbide *a* oxygenase (Pruzinska et al., 2003), TIC55 functions as monooxygenase that incorporates one oxygen atom derived from molecular oxygen into the chlorophyll catabolite pFCC (Hauenstein et al., 2016). The function of TIC55 as phyllobilin hydroxylase (Hauenstein et al., 2016) is clearly distinct from that in protein import originally described by Caliebe et al. (1997) and raises doubts on the existence of a redox regulon in chloroplasts, comprising TIC55, TIC32, and TIC62 and controlling protein import and plant viability (Benz et al., 2009; Boij et al., 2009; Balsera et al., 2010). While the role of TIC55 as phyllobilin hydroxylase seems meanwhile established that of phyllobilins and respective products remains elusive. Kräutler (2016) proposed phyllobilins to function as antioxidants, light filters and/or optical brighteners, while not precluding the idea of other, regulatory roles.

The plant non-heme oxygenases CAO and PAO participate in chlorophyll synthesis and degradation, respectively. Sequence alignments and hydrophilicity profiling (Supplementary Figures S6, S7) suggest that PTC52 is much more closely related to PAO than to CAO and it was therefore somewhat surprising to find that PTC52 exhibited a catalytic activity more similar to CAO and that the enzyme did not accept either Chlide *a* or phaeophorbide *a* as substrates. An examination of algal genomes revealed several PAO and PTC52-like genes but uncertainty in regards to gene splicing has not yet allowed the unambiguous discernment of PTC52 proteins in those genomes. Biochemical evidence supports the presence of a PTC52 function in *Chlamydomonas* and a CAO-like activity in the inner algal membrane (Bednarik and Hoober, 1985; Eggink et al., 2004).

Structural modeling was used to illuminate the issue of substrate specificity of PTC52. Using the published X-ray structure of KshA from *R. rhodochrous* as template (Penfield et al., 2014), several highly conserved domains and polypeptide chain folds/topologies were identified that comprise the Rieske and non-heme Fe binding domains and thioredoxin target signature (Figure 6A,B). In addition, the presence of a conserved central region (PAO domain cd03480) could be highlighted in PTC52 and KshA that shares similarity to the SRPBCC motif in the ligand-binding domain superfamily. In KshA, this central domain is comprised largely of antiparallel β-sheet within which 6 residues (Q204, S229, M239, D241, L243, and Y245) are directly involved in substrate binding in a so-called helix-grip fold (Figure 7B) (Petrusma et al., 2012; Penfield et al., 2014). In KshA residues N175 and I176 coordinate substrate binding in the vicinity of the non-heme Fe (Figure 7C). When we examined the corresponding region of PTC52 for residues that are conserved amongst PTC52, but not PAO, CAO, or TIC55 proteins (Supplementary Figure S6), 19 amino acid residues (of which 7 are aromatic) were identified in PTC52 that are likely to provide substrate specificity (Figure 7A and Supplementary Table S1). The predicted location of these residues in the PCT52 model reveals that several of them (L335, F337, W351, F353, and P354) are located within the last two strands of the antiparallel β-sheet region (Figure 7D), overlapping the region in KshA that has been shown to coordinate substrate binding (Figure 7B). The loop region between these two β-strands also harbors several residues (C339, I340, V342, and S347) unique to PTC52 as well as residues that are conserved in both PTC52 and PAO (P344, G345, and R348) (Figure 7D). On the other side of the predicted substrate binding region, several residues are conserved in PTC52 (L378, S380, L384, and H386) and surround D381 predicted to coordinate the non-heme Fe binding (Figure 7E). Other residues in this region that are invariant amongst PTC52, PAO, TIC55, and CAO, such as F297, P300, and P341 (Figure 7D), may have a role in overall domain folding that reflects a common evolutionary origin and substrate recognition for these proteins. The residues within this region that are conserved within individual PTC52, PAO, TIC55, and CAO groups but not between them (Supplementary Figure S6) provide a basis for the rational that they do bind different substrates. It is of particular note that two amino acid substitutions in this region (*acd1-20* S338F and *acd1-E2* G353R) conferred strong mutant phenotypes on the most closely related protein PAO in *A. thaliana* (Greenberg and Ausubel, 1993; Gray et al., 1997; Pruzinska et al., 2003). It is attractive to hypothesize that reduced phaeophorbide binding to the *acd1-20* S338F and *acd1-E2* G353R mutant proteins may be the cause of the lethal phenotype. The fact that PTC52 is most similar to PAO and shares more conserved residues in this region than to any of the other Rieske non-heme iron oxygenases family members further suggests that it may also bind a chlorophyll intermediate. Using *in vitro*-mutagenesis we demonstrate that replacing P354 by an Ala residues gave rise to a catalytically inactive PTC52 protein, supporting the idea of a central role of this region in substrate discrimination leading to Pchlide *a* binding. Additional domains such as the mononuclear Fe binding E/D^241^N^242^x_2_Dx_2_Hx_4_H^253^ domain obviously participate in Pchlide *a* binding, as revealed by our *in vitro*-mutagenesis study. Other regions of the presumed substrate binding pocket, such as the F^297^xA/PPC/V^301^ and C/N^339^xP^343^ (being referred to as motifs A and B in Supplementary Figure S7) may contribute to this high-fidelity binding. Deleting these domains from PTC52 in fact lowered PTC52’s Pchlide *a* oxygenase activity, presumably because of reduced Pchlide *a* binding (Table 3). Together, our mutagenesis studies support our 3D- homology model and provide a foundation for further investigations for insights into the substrate specificity of PTC52 and treated Rieske non-heme oxygenase family members.

### Role of PTC52 During Plant Development

Two approaches were undertaken to study the role of PTC52 *in planta*: (i) a genetic approach using T-DNA insertion mutants in the *PTC52* gene, and (ii) a DEX-inducible RNA interference (RNAi) approach. In our hand, attempts to obtain homozygous knock-out mutants for *PTC52* (SALK_011945 and Garlic_148_H05) failed presumably because of embryo lethality (Supplementary Figure S8). For another mutant allele (GK-043G03), viable plants were obtained (Rosso et al., 2003), but this mutant turned out to be a false-positive expressing unaltered levels of *PTC52* transcript and PTC52 protein (Supplementary Figure S8). In part, these results explain previous findings by Boij et al. (2009) who described that knock-out of the *PTC52* gene would not affect plant viability. Yet, the situation is unclear as Hauenstein et al. (2016) reported on a fourth *ptc52* mutant allele (SALK_006984) not analyzed here that was claimed to be viable. Because no data were presented by Hauenstein et al. (2016) to justify the homozygous nature of the mutant, care must be taken with regard to the published *PTC52* knock-out mutant genotypes and phenotypes. In our hands, self-crossed heterozygous *AtPTC52-1/Atptc52-1* and *AtPTC52-3/Atptc52-3* plants that were derived from SALK_011945 and Garlic_148_H05, respectively, did not produce viable homozygous seeds and aborted a fraction (≈25%) of their embryos at an early stage correlating with the formation of globular stages (Supplementary Figure S8). Segregation analyses for the T-DNA–linked kanamycin resistance gene provided a ratio of 66.3% resistance to 33.7% sensitive plants, a value that is very close to the expected 2:1 ratio if the knockout of *AtPTC52* was lethal (Supplementary Figure S8). Physiological and biochemical experiments on heterozygous *AtPTC52-1/Atptc52-1* and *AtPTC52-3/Atptc52-3* seedlings revealed perturbations in pigment accumulation that were caused by defects in PTC52-driven Pchlide *b* synthesis and binding to the PORA operating as light scavenger and conferring photoprotection during greening (Supplementary Figures S9, S10). All of these defects were overcome by genetically complementing *AtPTC52-1/Atptc52-1* and *AtPTC52-3/Atptc52-3* plants with the PTC52 cDNA (Supplementary Figure S11). In such genetically complemented plants, wild-type levels of Pchlide *a* and Pchlide *b* were restored (Supplementary Figure S12). DanePy measurements provided evidence for pigment-sensitized singlet oxygen formation as cause for the cell death phenotype (Supplementary Figure S13).

**FIGURE 8:**
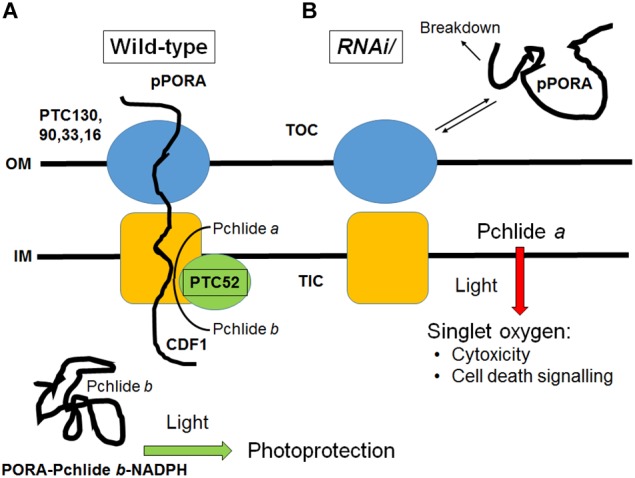
Working model describing the role of PTC52 *in planta*. **(A)** In wild-type plastids, PTC52 is active as Pchlide *a* oxygenase and converts Pchlide *a* to Pchlide *b* that in turn triggers the substrate-dependent import of pPORA into etioplasts. As a result, PORA-Pchlie *b*-NADPH complexes are formed that confer photoprotection onto greening seedlings. **(B)** The lack of PTC52 in DEX-induced *RNAi* plants and presumably also in homozygous *ptc52* mutants (not shown) leads to the uncontrolled accumulation of Pchlide *a* which, upon illumination, operates as photosensitizer and triggers the evolution of singlet oxygen. Given the documented role of singlet oxygen as cytotoxin and cell death signaling compound, seedling lethality is observed. The unimported pPORA is degraded in the cytosol. OM, outer plastid envelope, IM, inner plastid envelope; TOC, translocon at the outer plastid envelope; TIC, translocon at the inner plastid envelope. Note that the TOC and TIC machineries engaged by pPORA during import are distinct from the general TOC and TIC complexes used by photosynthetic precursors (Reinbothe et al., 2004a,b).

To overcome the difficulties encountered with the genetic approach not permitting to isolate homozygous plants deprived of PTC52, DEX-inducible RNA interference was used (Figure 5). Controls using RT-PCR and Western blotting confirmed the absence of *PTC52* transcript and PTC53 protein in the established *RNAi* lines (Figure 5 and Supplementary Figure S11). Pigment quantification revealed a severe deficiency in Pchlide *b* levels in DEX-induced *RNAi* seedlings in the dark and ca. 5-fold up-regulation in the amount of Pchlide *a* (Table 2). The observed imbalance in Pchlide homoeostasis provoked severe greening defects and led to cell death and seedling lethality (Figure 5) that were similar to those for heterozygous *AtPTC52-1/Atptc52-1* and *AtPTC52-3/Atptc52-3* at high light intensities (Supplementary Figure S13 and Supplementary Table S2). As mentioned above, a likely mechanism could be that Pchlide *b* needed for the substrate-dependent plastid import pathway of pPORA was absent from *PTC52 RNAi* plants (Figure 8). As a result of the PTC52 deficiency, large amounts of Pchlide *a* accumulated but could not be bound to protein. pPORA is known to be specific for Pchlide *b* and by binding the pigment confers photoprotection on dark-grown seedlings during greening (see Reinbothe et al., 2010b, for review). Free Pchlide *a* molecules accumulating in *RNAi* seedlings, by contrast, operated as photosensitizer and triggered the production of singlet oxygen causing cytotoxic and cell death signaling effects (Figure 8; see also Kim et al., 2008; Mochizuki et al., 2010; Reinbothe et al., 2010b; Wittkopp et al., 2017, for review). The finding that homozygous *ptc52* mutants were lethal indicates that PTC52 also plays an essential role in seed development. In Arabidopsis, the immature embryos and seeds undergo a greening process beginning in the outermost cells of the heart shaped embryo (Cairns et al., 2006). Chloroplasts present in these embryos (“chloroembryos”) are photosynthetically active and remain so through later embryo development before turning brown during the desiccation stage (Yakovlev and Zhukova, 1980; Puthur et al., 2013). The availability of expression atlas data (Le et al., 2010; Hofmann et al., 2019) reveals that the *PTC52* gene is indeed expressed in developing Arabidopsis seeds (seed coat, embryo, and chalazal endosperm) as early as the preglobular stage but starts reaching higher levels in the seed coat by the heart stage (Supplementary Figures S14, S15A,B). PORA is expressed at low levels in the embryo and at a slightly later developmental time point (Supplementary Figure S15C), whereas the co-chaperone CDF1 (cell growth defective factor 1) is expressed earlier and throughout embryo development until desiccation (Supplementary Figure S15D). Although the absolute expression levels of these three genes differ, their comparative expression is largely parallel and no more than 3.8-fold different at most (Supplementary Figures S15E,F). We had previously reported that CDF1 is another component operating in the Pchlide-dependent plastid import pathway of pPORA, (Lee et al., 2013; Reinbothe et al., 2015; Gray et al., 2016). Similar to *PTC52* knock-out mutants, CDF1 T-DNA insertion mutants were embryo-lethal (Lee et al., 2013; Reinbothe et al., 2015) and DEX-induced RNA interference of CDF1 caused the same porphyrin-sensitized cell death phenotype as observed here for *RNAi* seedlings deprived of PTC52. CDF1 is operative as a holdase to permit the interaction of the pPORA with its cognate substrate, Pchlide *b*, during protein import (Reinbothe et al., 2015). CDF1 and PTC52 [which we also previously demonstrated to be a redox sensor (Bartsch et al., 2008; Bartsch, 2009)], thus appear to be part of a common mechanism regulating pPORA import, Phlide homoeostasis and photoprotection *in planta*. Indeed, the initiation of photosynthetic tissue in the embryo is likely to involve a series of molecular events similar to that of de-etiolation for which PORA function is critical. In such a scenario, the absence of PTC52 or CDF1 is just as likely to result in a light-induced cell death cascade in embryos as in other stages of photosynthesis establishment. In this respect, it is not necessary to propose an alternative function for PTC52 in embryos but simply one that parallels its role in greening at later stages of plant development. It would be of interest to see if reduced light incidence on developing siliques may permit the recovery of homozygous *ptc52* seeds although these would be predicted to die during the de-etiolation process. Similarly, it would be hypothesized that in plants that do not possess a “chloroembryo” (i.e., maize) that a homozygous *ptc52* mutant would not be embryo-lethal.

### Evolution of *PTC52* and Related Genes

The detection of Pchlide *b* in higher plants has been a matter of dispute for a long time. The Bogorad laboratory originally formulated the concept of a branched chlorophyll biosynthetic pathway beginning at the stage of Pchlide *a* and leading to two independent routes of Chl(ide) *b* synthesis (Bogorad, 1967). With the identification of Pchlide *b* and *PTC52* genes and respective orthologs in different plant phyla and taxa (cf. Supplementary Figures S6, S7, see also Shedbalkar et al., 1991; Reinbothe S. et al., 2003; Kolossov and Rebeiz, 2004; Pollmann et al., 2007) and especially the detection of PTC52’s activity as Pchlide *a* oxygenase (Figure 1 and Table 1), these earlier results now find some new support. Confirming previous results (Reinbothe et al., 1999; Reinbothe S. et al., 2003; Buhr et al., 2008; see Reinbothe et al., 2010a, for review), Pchlide *b* was identified to be part of a photoprotective mechanism involving PORA. Hereby, PORA operates as light scavenger in etiolated seedlings undergoing greening (Buhr et al., 2008, 2017; Hanf et al., 2011; Yuan et al., 2012). It remains to be determined if this protective role underlies the presence of 4 tandem repeats of the PTC52 gene in the rice genome (Yu et al., 2002). The detection of a *PTC52-like* gene (Supplementary Figures S6, S7 and Gray et al., 2004) and a *POR* gene in *Synechocystis* (Suzuki and Bauer, 1995) supports the notion that in primordial plastids PTC52 may have served as porphyrin scavenger that evolved to sense the partial O_2_ pressure and to control the assembly of the photosynthetic membrane complexes containing POR (Reinbothe et al., 1996a,c, 2010a). In line with this notion, mutants of *Synechocystis* that lack POR are light-hypersensitive and impaired in normal photosynthetic function (He et al., 1998). Work is in progress to further study the role of PTC52 homologs in *Synechocystis* and higher plants and understand their function in redox regulation.

## Materials and Methods

### Plant Growth

Seeds of *A. thaliana* ecotype Columbia or barley (*Hordeum vulgare* cv. Carina) were germinated at 25°C and grown either in complete darkness or under continuous white light illumination provided by fluorescent bulbs (50 μE m^-2^ sec^-1^) for 4–5 days, as indicated. For inducing dexamethasone (DEX)-induced RNA interference (RNAi), seedlings were grown for 4 days in darkness, treated with DEX for 12 h or mock-incubated and either kept in the dark or exposed to white light of 125 μE m^-2^ sec^-1^ for 4 h (Reinbothe et al., 2015).

### DNA Constructs and Protein Expression

Full-length cDNA encoding *A. thaliana* transit peptide-containing or transit peptide-less forms of PTC52 (AtpPTC52 and AtPTC52, respectively) bearing hexa-histidine [(His)_6_] tags at their COOH termini were isolated as described (Gray et al., 2004). cDNA clones encoding the pPORA and pPORB of barley, the small subunit precursor of ribulose-1,5-bisphosphate carboxylase/oxygenase of soybean (SSU), precursor ferredoxin of *Silene pratensis* (FD) and the precursors to LHCII have been detailed elsewhere (Reinbothe et al., 2005, 2006). ^35^S-proteins were synthesized in wheat germ extracts by coupled *in vitro* transcription/translation or produced in *E. coli* and purified via Ni-NTA chromatography to 85–90% homogeneity. DNA sequencing used to confirm the identity of the generated clones was carried out by GATC Biotech AG (Constance, Germany).

### Measurement of PTC52 Activity

PTC52 activity was measured using established procedures, with either the *in vitro*-expressed, wheat germ-translated and purified At/HvpPTC52 and At/HvPTC52 proteins or functional PTC52-(His)_6_ envelope protein complexes as described (Reinbothe et al., 2004a; Bartsch et al., 2008). Final 50 μL-assays contained the following supplements: 2 mM Pchlide *a*, Chlide *a* or phaeophorbide *a*, 10 μg of FD (Sigma) and a FD-reducing system [2 mM glucose-6-phosphate; 1 mM NADPH; 50 milliunits (mU) of glucose-6-phosphate dehydrogenase; 10 milliunits (mU) of FD-NADPH-oxidoreductase (FNR) (Sigma)] (modified after Oster et al., 2000; Pruzinska et al., 2003). Final PTC52 protein concentrations for *in vitro*-activity measurements were 60–100 μg/mL. Activity measurements with phaeophorbide *a* (Reinbothe et al., 2006; Bartsch et al., 2008) included *in vitro*-expressed red chlorophyll catabolite reductase (RCCR) from *A. thaliana* (Pruzinska et al., 2003).

### Pigment Measurements

Pigments were extracted with 100% acetone containing 0.1% diethyylpyrocarbonate (DEPC) and separated by HPLC on a C18 reverse phase silica gel column (Shandon, Hypersil ODS, 5 μm), using synthetic Pchlides *a* and *b* as references (Reinbothe C. et al., 2003; Reinbothe S. et al., 2003). Pigment identification and quantification were made at 455 nm, using a photodiode array detector. Mass spectroscopy of HPLC-separated pigments was performed by matrix-assisted laser desorption/ionization time-of-flight mass spectrometry (Voyager DE STR Biospectrometry Work Station, Foster City, CA, United States). As reference, 10 μg of Pchlide *b* was mixed with terthiophene (used as matrix) dissolved in acetone.

### Plastid Isolation, Manipulation, and Protein Import

Plastids were isolated from surface-sterilized leaf tissues by differential centrifugation and Percoll/sucrose density centrifugation (Reinbothe et al., 1995b). Protein import was studied in 50-μL import mixtures consisting of 25 μL of doubly concentrated import buffer, 10 μL of Percoll-purified, intact, energy-depleted, resuspended plastids (5 ⋅ 10^7^), and 5 μl of urea-denatured, radiolabeled precursors (final 0.2 M urea concentration). Mg-ATP and Mg-GTP were added to the final concentrations indicated in the text (Reinbothe et al., 2004a). For the experiment described in Figure 4, the assays were supplemented with a solution of DEPC to provide the indicated final inhibitor concentrations. If needed, doubly distilled water was added to adjust the final reaction volume. All assay mixtures were assembled on ice under a dim green safe-light; the actual import reactions were performed at 23°C for 15 min in darkness. Post-import protease treatment of reisolated plastids was performed with thermolysin (Cline et al., 1984). Salt extraction of membranes was achieved with either 0.1 M sodium carbonate, pH 11, or 1 M NaCl (Cline et al., 1984). Plastid fractionation into envelopes, stroma and thylakoids was carried out according to Li et al. (1991) or Schnell et al. (1994). Protein was precipitated with trichloroacetic acid (TCA) [5% (w/v) final concentration] and resolved by SDS-PAGE on 10–20% (w/v) polyacrylamide gradients (Laemmli, 1970). Protein complexes of AtPTC52-(His)_6_ were isolated from detergent-solubilized envelope membranes of ruptured chloroplasts by Ni-NTA chromatography and separated by non-denaturing PAGE (Reinbothe et al., 1990, 2004b). Proteins were detected by autoradiography. Two-dimensional separations of proteins included isoelectric focusing in the first dimension and SDS-PAGE in the second dimension (Scharf and Nover, 1982).

### Dexamethasone-Induced RNA Interference

DNA cloning and construction of RNAi lines was made as described by Lee et al. (2013). Plant transformants were identified by PCR (Innis et al., 1990) and Southern blotting (Sambrook et al., 1989), using appropriate primers and probes, respectively.

### Seedling Viability Tests

Seedling viability was assessed by tetrazolium staining (Nortin, 1966). While vital seedlings show a strong red staining, dead seedlings are unable to retain the dye and look yellow-whitish. For statistic assessment, pools of about 250 seeds were analyzed in three independent experiments.

### Singlet Oxygen Measurements

Singlet oxygen generation was measured with DanePy (Hideg et al., 1998; Kálai et al., 2002). Fluorescence emission of DanePy was collected between 425 and 625 nm, using a Life Sciences spectrometer LS50 (Perkin Elmer Corp., Norwalk, CT, United States) an excitation wavelength of 330 nm.

### Immunological Techniques

Immunoprecipitation was carried out according to Wiedmann et al. (1987), using the antisera specified in the text. Western blotting was done according to Towbin et al. (1979), using anti-rabbit, anti-goat, alkaline phosphatase-based or enhanced chemiluminescence (ECL)-based (Amersham-Pharmacia) detection systems.

### Bioinformatics Tools

Biocomputational methods and sequence data sources are described in the Supplementary Information section. Gene expression data were retrieved from The Bio-Analytic Resource for Biology (BAR) http://www.bar.utoronto.ca/.

## Author Contributions

SR designed research, carried out experiments, analyzed data, and wrote the manuscript. SB, ClR, and MD carried out research and analyzed data. SY designed research, carried out research, and analyzed data. ChR designed research, carried out research, analyzed data, and wrote the manuscript. JG carried out research, analyzed data, and wrote the manuscript.

## Conflict of Interest Statement

The authors declare that the research was conducted in the absence of any commercial or financial relationships that could be construed as a potential conflict of interest.

## Footnotes

1 www.rcsb.org
