# Supplementary material for: A Protochlorophyllide (Pchlide) a Oxygenase for Plant Viability
Source: Front Plant Sci. 2019 May 15;10:593. doi: 10.3389/fpls.2019.00593 (PMC6530659; doi:10.3389/fpls.2019.00593)
Supplement: Supplementary file 1 [file Data_Sheet_1.pdf]

Supplementary Data

Supplementary Methods

PTC52 knock-out mutants and *RNAi* lines of *A. thaliana*

T-DNA insertion lines in the *AtPTC52* gene (At4g26560) were identified in the T-DNA collection of the Salk Institute Genome Analysis Laboratory (SALK_011945; Alonso et al., 2003), Genomanalyse im Biologischen System Pflanze-Kölner *Arabidopsis* T-DNA (GABI-Kat) stock center (GK-043G03; Rosso et al., 2003) and Syngenta collection (Garlic _148_H05.b.1a.Lb3Fa; identical with SAIL_148.HC5; Sessions et al., 2002)(referred to as *Atptc52-1, 3/Atptc52-2* and *Atptc52-3* in Fig. S8), using a reverse transcription-polymerase chain reaction (RT-PCR)-based approach with specific primers (Innis et al. 1990). Dexamethason (DEX)-induced RNA interference (*RNAi*) was carried out according to Lee at al., 2013).

Genetic complementation and dexamethasone-induced RNA interference

DNA cloning was performed following Gateway Technology (Invitrogen), as described (Samol et al., 2011a, 2011b). Briefly, cDNAs for AtPTC52 was integrated into the binary T-DNA destination vectors pB7WG2, pK7WGT2 and pB7YWG2 to generate the following cDNA constructs: 35S::PTC52, 35S::GFP::PTC52, and 35S::PTC52::YFP. In case of 35S::PTC52::YFP, the stop codon of the *AtPTC52* cDNA was eliminated during PCR cloning using appropriate primers (Innis et al., 1990). These generated constructs in turn were transform into *AtPTC52-1/Atptc52-1* and *AtPTC52-3/Atptc52-3* plants by the floral dip method (Clough and Bent, 1998). Plant transformants were identified by PCR (Innis et al., 1990) and Southern blotting (Sambrook et al., 1989), using appropriate primers and probes, respectively. To further study the impact of PTC52 deficiency *in vivo*, we used dexamethason-inducible RNA interference, as described by Lee et al. (2013).

Plant growth conditions and physiological tests

Seeds of heterozygous and ecotype Columbia were germinated at 25°C and grown either in complete darkness or under continuous white light illumination provided by fluorescent bulbs (50 µE m^{-2} sec^{-1}) for 4-5 days, as indicated. For illumination experiments, 4 d-old seeds were exposed to low (5 µE m^{-2} sec^{-1}, medium (125 µE m^{-2} sec^{-1}), and high (250 µE m^{-2} sec^{-1}) intensities of white light for variable periods. Seedling viability was assessed by tetrazolium staining (Nortin, 1966). For DEX-induced RNAi, seedlings were grown for 4 d in darkness, treated with DEX for 12 h or mock-incubated and either kept in the dark or exposed to white light of 125 µE m^{-2} sec^{-1} for 24 h (Reinbothe et al., 2015).

Biochemical assays

Protein gel blot analyses were carried out according to Towbin et al. (1979) using the indicated antisera. For electrophoretic separation, 10-20% polyacrylamide (PAA) gradients containing SDS or SDS-containing 15% PAA mini-gels were used. For detection of POR super-complexes, non-denaturing PAA gel electrophoresis was employed (Reinbothe et al., 1990). Conditions for studying protein uptake are highlighted in the main text. Pigment identification and quantification were performed by HPLC, using established procedures (Reinbothe et al., 2003a, 2003b). Quick-step chlorophyll measurements were made according to Porra (1989). Singlet oxygen production was quantified using DanePy (Hideg et al., 1998; Kálai et al., 2002).

Biocomputational methods and sequence data sources

Multiple sequence alignments were performed using Clustal W method with Gonnet 250 residue weights within the Megalign® program (DNAStar, Madison, WI). Unrooted phylograms were generated by using the PAUP 4.0a (build 161) for Macintosh program (paup.phylosolutions.com) (Swofford, 2002). The bootstrap method was performed for 1000 replicates with a maximum parsimony criterion. All characters were weighted equally. Starting trees were obtained by random stepwise addition and the tree-bisection-reconnection algorithm was used for branch swapping. Amino acid sequences of proteins were predicted from available GenBank files and genome databases (www.phytozome.net and www.maizegdb.org). Predicted proteins were supported in most cases by full length cDNAs and ESTs and in a few instances aligned ESTs were used to correct the predicted protein. GenBank Accession numbers are listed below and where conceptual translation was used the nucleotide co-ordinates are also supplied: *At* (*Arabidopsis thaliana*) *CAO* (AAD54323), *AtPAO* (NP_190074.1), *AtTIC55* (NP_180055.1), *AtPTC52* (NP_567725.1); *Os* (*Oryza sativa*) *CAO* (NP_001065433.1), *OsPAO* (EAY88539.1), *OsTIC55* (NP_001048363.10, *OsPTC52-1* (NM_0010581650; *Pp* (*Physcomitrella patens*) *CAO-2* (XP_001777722), *PpPAO-1* (XP_001781796.1), *PpTIC55-1* (XP_001772643.1), *PpPTC52* (XP_001753171.1); *Zm* (*Zea mays*) *CAO-2* (AC186790 77334..77408, 77636..77878, 77994..78098, 78210..78494, 78596..78743, 79088..79272, 79363..79475, 79571..79865, 79981..80163), *ZmPAO* (AAC49678.1), *ZmTIC55* (AC186589.3 95605..95211, 95100..94998, 94904..93786), *ZmPTC52* (AC201906 100194..99500, 99359..99259, 98836..98694, 98565..98365, 98228..98041, 97936..97678); *N.*sp (*Nostoc* sp. PCC 7120) ALR4354 (NP_488394.1), *N.*sp ALR5007 (NP_489047.1|); *Np* (*Nostoc punctiforme* PCC 73102) F1863 (YP_001865463.1); *Te* (*Trichodesmium erythraeum* IMS101) P1779 (ZP_00072570); and *Synechocystis* sp. PCC 6803 SLR1747 (NP_441106). Naphthalene dioxygenase (NahAc) sequence was from *Pseudomonas putida* strain G7 (JN0644). Proteins from *Populus trichocarpa* (*Pt*) were identified using BLAST against the complete genome (JGI v1.1 at www.phytozome.net V3.0). The coordinates of these genes are *Pt*CAO-1 (linkage group V, 11564764..11564841,5803690..5803448,11565753..11565857,11566086..11566361,11566581..11566728,11566834..11567019,11567142..11567254,11567802..11568096,11568251..11568421), *Pt*PAO-1 (Scaffold 163, 245278..244864,244482..244243,244126..243996,243079..242939,242320..242157, 240835..240551,240203..239945), *PtTIC55*-1 (linkage group XVIII, 6507907..6507476,6507175..6507069,6506905..6505781) and *PtPTC52* (linkage group I, 399368..398831,398456..398259, 397953..397866,397796..397608,397433..397242,396688..396492,396417..396156).

SI Results

Evolutionary origin of PTC52 and related Rieske proteins

A comprehensive bioinformatics study was carried out to understand the possible evolutionary origin of PTC52, PAO, CAO, and TIC55. Figures S6 and S7 suggest that PTC52, PAO, CAO, and TIC55 evolved from a cyanobacterial ancestral gene (similar to alr4345 from *Nostoc* sp PCC 7120) whose function may have evolved during the transition to oxygenic photosynthesis (see also Gray et al., 2004). In contrast, the CMO gene appears to have a separate origin and is more closely related to enzymes in soil bacteria that catabolize aromatic compounds (Gray et al., 2004). In addition to the highly conserved Rieske ($CxHx_{16-17}Cx_2H$) and mononuclear iron binding ($Nx_2Dx_{3-4}Hx_4H$) motifs, PTC52, PAO and TIC55 proteins from different plant species share the presence of a conserved, CxxC motif reminiscent of thiolreductases at approximately 73 amino acids from the carboxy terminus which is also found in homologs from *Nostoc* and other cyanobacteria (Figs S6 and S7). Via this CxxC motif, TIC55, PTC52 and PAO are prone to regulation by the thioredoxin system and also respond to oxidative stress (Bartsch et al., 2008). No CxxC

motif is present in CAO, which exhibits a larger NH_2-terminus not contained in PTC52, PAO or TIC55, and CAO does not respond to either the thioredoxin system or oxidative stress (Bartsch et al., 2008). Using *in planta* assays, Yamasoto et al. (2005) showed that the NH_2-terminus of CAO serves as Chlide *a* sensor and regulates the stability of the enzyme.

Identification of *Atptc52* knock-out plants

Arabidopsis mutants were identified in the Salk Institute Genomic Analysis Laboratory collecætion (Alonso et al., 2003) carrying a T-DNA insertion "-198" bp upstream of the ATG start codon of the *AtPTC52* (At4g25650) gene (SALK_011945)(Fig. S8A). The other *Atptc52-1* mutants ,comprised a T-DNA insertion line (GK-043G03; referred to as *Atptc52-2* in Fig. S8A) from the Genomanalyse im Biologischen System Pflanze-Kölner *Arabidopsis* T-DNA (GABI-Kat) stock center (Rosso et al., 2003) and a T-DNA insertion line from the Syngenta collection (Garlic_148_H05.b.1a.Lb3Fa, SAIL_148.HC5; referred to as *Atptc52-3* in Fig. S8A; Sessions et al., 2002) of which *Atptc52-2* turned out to be a false-positive mutant carrying the T-DNA insertion "-97" bp in front of the ATG start codon and not, as indicated by the supplier, in exon 1. Homozygous knockout plants were obtained in the progeny of self-crossed *AtPTC52-2/Atpc52-2* plants that still contained wild-type levels of PTC52 protein (Fig. S8B). Contrary to *AtPTC52-2/Atpc52-2,* the progeny of self-crossed *AtPTC52-1/Atpc52-1* and *AtPTC52-3/Atpc52-3* plants did not contain such homozygotes, suggesting that mutation of the *AtPTC52* locus is lethal. Segregation of the T-DNA–linked kanamycin resistance gene produced a ratio of 66.3% resistance to 33.7% sensitive plants, a value very close to the expected 2:1 ratio if the knockout of *AtPTC52* was lethal and prevented the production of viable seeds. Visual analysis of the siliques of self-crossed *AtPTC52-1/Atpc52-1* plants (Fig. S8C, panel a) indicated that approximately one-quarter (24.5%) of the seeds failed to develop. Microscopic analyses revealed that embryo abortion occurred at an early, globular stage (Fig. S8C, panel b). Both, heterozygous *PTC52-1/ptc52-1* and At*PTC52-3/Atptc52-3* plants contained reduced amounts of PTC52 protein (Fig. S8B).

Haplo-insufficiency of PTC52 in heterozygous *AtPTC52-1/Atptc52-1* and *AtPTC52-3/Atptc52-3* plants

Heterozygous *AtPTC52-1/Atptc52-1* and *AtPTC52-3/Atptc52-3* plants were utilized to gain insights into the role of PTC52 *in planta*. *AtPTC52-1/Atptc52-1* seeds we germinated in the dark for variable periods and subsequently exposed to white light. Visual inspection of the seedlings suggested an impairment of greening (data not shown). The greening defect was dependent on seedling age. Younger *AtPTC52-1/Atptc52-1* seedlings were less sensitive to illumination than older seedlings (data not shown). Chlorophyll accumulation kinetics confirmed that *AtPTC52-1/Atptc52-1* seedlings greened more slowly than wild-type plants at low light intensities (5 µE m^{-2} sec^{-1})(data not shown) as well as medium light (125 µE m^{-2} sec^{-1}) intensities (Fig. S9 and Table S2). At high light intensities (250 µE m^{-2} sec^{-1}), a significant fraction of *AtPTC52-1/Atptc52-1* died upon a dark-to-light shift (Table S2). A similar light intensity effect on greening and seedling viability was obtained for *AtPTC52-3/Atptc52-3* (Fig. S10 and data not shown). *AtPTC52-1/Atptc52-1* and *AtPTC52-3/Atptc52-3* expressed ≈40% and ≈10%, respectively, of the total chlorophyll content found in the wild-type after 3 days of greening (Fig. ~~S5 and S6~~S2 and S3). When the abundance of NADPH:protochlorophyllide oxidoreductase (POR) was examined by Western blotting, two bands were visible in etioplast protein extracts, of which the upper band was barely detectable in 5 d-old etiolated *AtPTC52-1/Atptc52-1* and *AtPTC52-3/Atptc52-3* seedlings, as compared to wild-type seedlings (Fig. S9 and S10, panel Ba each). In 4 d-old etiolated *AtPTC52-1/Atptc52-1* and *AtPTC52-3/Atptc52-3* seedlings that had been exposed to white light of 125 µE m^{-2} sec^{-1} for 24 h, both POR protein bands were below the limit of detection, while only the upper

band was reduced in amount in the wild-type (Fig. S9 and S10, panel Ba). We interpret this result as evidence for the specific lack of PORA in the dark in *AtPTC52-1/Atptc52-1* and *AtPTC52-3/Atptc52-3* seedlings and a superimposed light effect on PORB during greening. It has been shown that the PORA and PORB form larger complexes in barley (Reinbothe et al., 1999; 2003; Yuan et al., 2012) and Arabidopsis (Buhr et al., 2017) and that lack of one POR protein affects the abundance and function of its respective partner (Buhr et al., 2008). Control Western blot studies using protein extracts from isolated etioplasts of *pora*- and *porb* knock-out plants (Samol et al., 2011b) confirmed the identity of the two POR protein bands as PORA (upper band) and PORB (lower band)(Fig. S9Ba). Non-denaturing electrophoresis of POR-pigment complexes confirmed drastically reduced amounts of light-harvesting POR-Pchlide (LHPP) complexes containing the PORA and PORB in the prolamellar bodies of etioplasts in *AtPTC52-1/Atptc52-1* and *AtPTC52-3/Atptc52-3,* as compared to wild-type, seedlings (Fig. S9 and S10, panel C). Pigment analyses by HPLC identified both Pchlide *a* and Pchlide *b* in wild-type seedlings and unveiled a change in their relative proportion towards Pchlide *a* in *AtPTC52-1/Atptc52-1* seedlings (Fig. S9D). These changes correlated with an altered Chl *b*-to-Chl *a* ratio and a reduction of LHCII levels during the early hours of greening (Fig. S9B, panel b, and Table S2). Together, the results suggested decreases in normal Chl accumulation that were caused by the haplo-insufficiency of PTC52 in heterozygous *PTC52* knock-out mutants. *In vitro*-import experiments demonstrated ≈90% reductions in uptake of ^{35}S-pPORA, but not of ^{35}S-pPORB, into chloroplasts of *AtPTC52-1/Atptc52-1* plants (Fig. S9E). Import experiments carried out with ^{35}S-pSSU of soybean and ^{35}S-pre FD of *Silene pratensis* (pFD) did not reveal gross differences for *AtPTC52-1/Atptc52-1* and wild-type chloroplasts (Fig. S9E). By contrast, ≈40-50% reductions in import of ^{35}S-pLHCII were observed for *AtPTC52-1/Atptc52-1* versus wild-type chloroplasts, suggesting some redundant function of PTC52 in pPORA and pLHCII import. As previously proposed (Reinbothe et al., 2006), LHCII requires with Chlide *b* the product of the PORA reaction that was impeded by the reduction in the amount of substrate (Pchlide *b*) in the *AtPTC52-1/Atptc52-1* mutant.

Genetic complementation of *AtPTC52-1/Atptc52-1* and *AtPTC52-3/Atptc52-3*

Complementation assays were used to further assess the role of PTC52 *in planta*. Heterozygous *AtPTC52-1/Atptc52-1* and *AtPTC52-3/Atptc52-3* seedlings were transformed with the AtPTC52 cDNA constructs in the Materials and Methods section above. After transformation by the floral dip method (Clough and Bent, 1998), BASTA-resistant plants were identified and propagated further. Upon Southern blotting, only lines containing singlet DNA inserts were selected and characterized further (Buhr et al., 2017). RT-PCR analyses and Western blotting confirmed that the identified transformed lines expressed *PTC52* transcript and PTC52 protein to wild-type levels, while *RNAi* plant sed for comparison were deprived of both (Figs. S11A and B). Most notably, genetically complemented *AtPTC52-1/Atptc52-1* and *AtPTC52-3/Atptc52-3* seedlings were viable, whereas untransformed *AtPTC52-3/Atptc52-3* and RNAi plants were not (Fig. S11C). Pigment measurements confirmed the restoration of wild-type levels of Pchlide *a* and Pchlide *b* (Fig. S12) and concomitant failure to produce singlet oxygen upon non-permissive dark-to-light shifts at 250 µE m^{-2} sec^{-1} (Fig. S13).

Expression of PTC52 over plant development

AtPTC52 transcript accumulation was followed by RT-PCR analyses and Northern blotting and turned out to be light-dependent and reduced in *AtPTC52-1/Atptc52-1* seedlings, as compared with wild-type seedlings (Fig. S14A and C). *AtPTC52* transcript levels remained low in leaves of 6-weeks-old *AtPTC52-1/Atptc52-1* plants (Fig. S14B), while no *AtPTC52* transcript was detectable in roots of *AtPTC52-*

1/Atptc52-1 and wild-type plants (Fig. S14B). RT-PCR analyses allowed demonstrating a transient rise in *AtPTC52* transcript abundance during early embryo development (globular stage)(Fig. S14D). Given the expected low amounts of PTC52 protein in the bulk of other silique proteins, no effort was made, however, to confirm respective changes in PTC52 protein levels (data not shown).

SI Discussion

Role of PTC52 during plant development

A genetic approach was undertaken to study the role of the *PTC52* gene *in planta*. In our hands, for two of the three *PTC52* mutant alleles studied no homozygous *Atptc52/Atptc52* plants could be obtained. This result is in part at clear variance with findings of Boij et al. (2009) who used the same SALK_011945 (*Atptc52-1)* and GK-043G03 (*Atptc52-2*) mutant alleles as we explored in this report but in either case did obtain homozygous plants. In our hands, no homozygous plants could be rescued for line SALK_011945 and only heterozygotes were found in the progeny of self-crossed *AtPTC52-1/Atptc52-1* plants. By contrast, line GK-043G03 at first glance seemed to produce homozygous *Atptc52-2/Atptc52-2*) plants. However, line GK-043G03 was found to contain the T-DNA insertion at "-97" bp upstream of the ATG start codon and thus at a location which is different from the predicted one. Homozygous GK-043G03 plants produced normal *AtPTC52* transcript and AtPTC52 protein levels, explaining why they were viable. By contrast, SALK_011945 did not provide viable plants, as assessed by segregation analyses for the resistance gene and respective genotyping of a large number of seeds. The reasons for the discrepancy with regard to the data by Boij et al. (2009) are unclear and need to be explored in future work. The fact that a second, independent embryo-lethal mutant allele was isolated in terms of Garlic_148_H05, the segregation and phenotypic properties of which were identical to those found for SALK_011945, nevertheless argues against the possibility of an artifact. When heterozygous *AtPTC52-1/Atptc52-1* and *AtPTC52-3/Atptc52-3* seedlings were grown for 4 d in darkness and exposed to strong white light (250 µE m^{-2} sec^{-1}) for 24 h, a significant fraction (≈85%) of the seedling population died. Presumably as a result of excess Pchlide *a* accumulation occurring in *AtPTC52-1/Atptc52-1* and *AtPTC52-3/Atptc52-3* seedlings, significant amounts of singlet oxygen were produced and caused cytotoxic and cell death signaling effects (see Reinbothe et al., 2010, for review). At low and medim light intensities (5 and 125 µE m^{-2} sec^{-1}, respectively), viable *AtPTC52-1/Atptc52-1* and *AtPTC52-3/Atptc52-3* seedlings could be rescued and grown to maturity. These seedlings nevertheless displayed some delay in greening at medium light intensities (125 µE m^{-2} sec^{-1}) and developed more slowly than wild-type plants most likely due to the haplo-insufficiency of PTC52 *in planta*. Dark-grown *AtPTC52-1/Atptc52-1* and *AtPTC52-3/Atptc52-3* seedlings lacked PORA operating as light scavenger during greening and suffered from photooxidative damage provoking cell death and seedling lethality at high light intensities (250 µE m^{-2} sec^{-1}). Using dexamethasone-induced RNA interference depriving PTC52 transcript and protein from dark-grown seedlings, even stronger effects were obtained that led to the distinction of the whole seedling population after dark-to-light shifts at 125 µE m^{-2} sec^{-1}. In genetically complemented *AtPTC52-1/Atptc52-1* and *AtPTC52-3/Atptc52-3* seedlings, wild-type *AtPTC52* transcript and AtPTC52 protein as well as Pchlide *a* and Pchlide *b* levels were restored and no light-dependent cell death occurred during greening. Together, these data provide strong evidence for an essential role of PTC52 during plants development.

How can the embryo-lethal phenotype of *Atptc52-1/Atptc52-1* and *Atptc52-3/Atptc52-3* be explained? Conceptually, embryo lethality may result from defects in key structural components and biochemical pathways needed for proper embryo development or perturbations in respective key

regulatory networks. The fact that PTC52 is an abundant plastid envelope protein in barley and Arabidopsis and displays Pchlide *a*-oxygenase activity is in favour of a structural and catalytic role. Because Arabidopsis and other angiosperms pass a period of transient photosynthesis activity during embryogenesis and seed development (Yakovlev and Zhukova, 1980; Medford and Sussex, 1989; Fyk et al., 1995), it is likely that they synthesize their Chl *b* via a reaction comprising PTC52 (Pchlide *a* to Pchlide *b* conversion) and PORA (reduction of Pchlide *b* to Chlide *b*). Lack to carry out this coupled reaction would perturb embryogenesis and cause embryo abortion, as encountered in siliques of self-crossed *AtPTC52-1/Atptc52-1* and *AtPTC52-3/Atptc52-3* plants. RT-PCR data confirmed PTC52 transcript accumulation in siliques during early embryogenesis such that PTC52 can be assumed to play a role as catalytic component and factor coupling Pchlide *b* synthesis to pPORA import during this particular window of plant development. At later stages, PTC52 transcript levels decline to low levels but remain nevertheless detectable in seeds to permit seed germination and greening. The observed biphasic temporal pattern of PTC52 expression seems to satisfactorily explain the phenotypic effects seen for PTC52 knock-out mutants and *RNAi* plants.

References

Alonso, J.M., Stepanova, A.N., Leisse, T.J., Kim, C.J., Chen, H., Shinn, P., Stevenson, D.K., Zimmerman, J., Barajas, P., Cheuk, R. *et al.* (2003) Genome-wide insertional mutagenesis of *Arabidopsis thaliana*. *Science* **301**, 653-657.

Bartsch, S., Monnet, J., Selbach, K., Quigley, F., Gray, J., von Wettstein, D., Reinbothe, S., Reinbothe, C. (2008) Three new thioredoxin targets in the inner plastid envelope membrane function in protein import and chlorophyll metabolism. *Proc. Natl. Acad. Sci. USA* **105**, 4933-4938.

Boij, P., Patel, R., Garcia, C., Jarvis, P, and Aronsson, H. (2009) In vivo studies on the roles of Tic55-related proteins in chloroplast protein import in *Arabidopsis thaliana*. *Mol. Plant* **2**, 1397–1409.

Buhr F, El Bakkouri M, Valdez O, Pollmann S, Lebedev N, Reinbothe S, Reinbothe C (2008) Photoprotective role of NADPH:protochlorophyllide oxidoreductase A. *Proc Natl Acad Sci USA* **105**(34), 12629-12634.

Buhr, F., Lahroussi, A., Springer, A., Rustgi, S., von Wettstein, D., Reinbothe, C., and Reinbothe, S. (2017) NADPH:protochlorophyllide oxidoreductase B (PORB) action in *Arabidopsis thaliana* revisited through transgenic expression of engineered barley PORB mutant proteins. *Plant Mol. Biol.* **94**(1-2), 45-59.

Clough, S., and Bent, A. (1998) Floral dip: a simplified method for *Agrobacterium*-mediated transformation of *Arabidopsis thaliana*. *Plant J.* **16**, 735-743.

Fyk, B., Bednara, J., and Rodkiewicz, B. (1995) Chlorophyll autofluorescence in globular and heart-shaped embryos of some dicotyledons. *Acta Soc. Bot. Pol.* **65**, 161-166.

Gray, J., Wardzala, E., Yang, M., Reinbothe, S., Haller, S., and Pauli, F. (2004) A small family of LLS1-related non-heme oxygenases in plants with an origin amongst oxygenic photosynthesizers. *Plant Mol. Biol.* **54**, 39-54.

Hellens, R.P., Edwards, E.A., Leyland, N.R., Bean, S., and Mullineaux, P.M. (2000) pGreen: a versatile and flexible binary Ti vector for Agrobacterium-mediated plant transformation. *Plant Mol. Biol*. **42**(6), 819-832.

Hideg, E., Kálai, T., Hideg, K., and Vass, I. (1998) Photoinhibition of photosynthesis *in vivo* results in singlet oxygen production detection via nitroxide-induced fluorescence quenching in broad bean leaves. *Biochemistry* **37,** 11405-11411.

Innis, M.A., Gelfand, D.H., Sninsky, J.J., and White, T.J. (1990) *PCR Protocols* (Academic Press, San Diego, CA).

Kálai, T., Hankovszky, O., Hideg, E., Jeko, J., and Hideg, K. (2002) Synthesis and structure optimization of double (fluorescent and spin) sensor molecules. *ARKIVOC* **iii**, 112-120.

Lee J-Y, Lee H–S Song J-Y Jung YJ, Park Y-I Lee SY Reinbothe S, Pai H-S (2013) CHAPERONE-LIKE PROTEIN OF POR1 plays a role in stabilization of light-dependent protochlorophyllide oxidoreductase in *Nicotiana benthamiana* and *Arabidopsis*. *The Plant Cell* 25, 1-17

Medford, J.I., and Sussex, I.M. (1989) Regulation of chlorophyll and RuBisCo levels in embryonic cotyledons of *Phaseolus vulgaris*. *Planta* **179**, 309-315.

Nortin, J.D. (1966) Testing of plum pollen viability with tetrazolium salts. Proc. *Amer. Soc. Hort. Sci.* **89,** 132-134.

Porra, R.J., Thompson, W.A., and Kriedemann, P.E. (1989) Determination of accurate extinction coefficients and simultaneous equations for assaying chlorophylls *a* and *b* extracted with four different solvents: verification of the concentration of chlorophyll standards by atomic absorption spectroscopy. *Biochim. Biophys. Acta* **975**, 384-394

Reinbothe, C., Bartsch, S., Eggink, L.L., Hoober, J.K., Brusslan, I., Andrade-Paz, R., Monnet, J., and Reinbothe, S. (2006) A role for chlorophyllide *a* oxygenase in the regulated import and stabilization of light-harvesting chlorophyll *a*/*b* proteins in chloroplasts. *Proc. Natl. Acad. Sci. USA* **103**, 4777-4782.

Reinbothe, C., Buhr, F., Pollmann, S., and Reinbothe, S. (2003) *In vitro*-reconstitution of LHPP with protochlorophyllides *a* and *b*. *J. Biol. Chem.* **278**, 807-815.

Reinbothe, C., Lebedev, N., and Reinbothe, S. (1999) A protochlorophyllide light-harvesting complex involved in de-etiolation of higher plants. *Nature* **397**, 80-84.

Reinbothe, C., Pollmann, S., and Reinbothe, S. (2010) Singlet oxygen signaling links photosynthesis to translation and plant growth. *Trends Plant Sci*. **15(9)**, 499-506

Reinbothe, S., Gray, J., Rustgi, S., von Wettstein, D., Reinbothe, C. (2015) Cell growth defect factor 1 is crucial for the plastid import of NADPH:protochlorophyllide oxidoreductase A in Arabidopsis thaliana. *Proc. Natl. Acad. Sci. USA* **112**(18), 5838-5343.

Reinbothe, S., Krauspe, R., and Parthier, B. (1990) Partial purification and characterization of mRNAs for chloroplast and cytoplasmic aminoacyl-tRNA synthetases from *Euglena gracilis*. *J. Plant Physiol.* ***137***, 81-87.

Reinbothe, S., Pollmann, S., and Reinbothe, C. (2003) *In-situ*-conversion of protochlorophyllide *b* to protochloro-phyllide *a* in barley: Evidence for a role of 7-formyl reductase in the prolamellar body of etioplasts. *J. Biol. Chem.* **278**, 800-806.

Rosso, M.G., Li, Y., Strizhov, N., Reiss, B., Dekker, K., and Weisshaar, B. (2003) An *Arabidopsis thaliana* T-DNA mutagenized population (GABI-Kat) for flanking sequence tag-based reverse genetics. *Plant Mol. Biol.* **53**, 247–259.

Sambrook, J., Fritsch, E., and Maniatis. T. (1989) *Molecular cloning. A laboratory manual*, 2nd edn. (Cold Spring Harbor Laboratory Press, New York).

Samol, I., Buhr, F., Springer, A., Pollmann, S., Lahroussi, A., Rossig, C., von Wettstein, D., Reinbothe, C., Reinbothe, S. (2011a) Implication of the *oep16-1* mutation *in* a *flu*-independent, singlet oxygen-regulated cell death pathway in *Arabidopsis thaliana*. *Plant Cell Physiol.* **52**, 84-95.

Samol, I., Rossig, C., Buhr, F., Springer, A., Pollmann, S., Lahroussi, A., von Wettstein, D., Reinbothe, C., Reinbothe, S. (2011b) The outer chloroplast envelope protein OEP16-1 for plastid import of NADPH:protochlorophyllide oxidoreductase A in Arabidopsis thaliana. *Plant Cell Physiol.* **52**, 96-

111.

Sessions, A., Burke, E., Presting, G., Aux, G., McElver, J., Dietrich, B., Ho, P., Bacwaden, J., Ko, C. *et al.* (2002) A high-throughput *Arabidopsis* reverse genetics system. *Plant Cell* **14**, 2985-2994.

Swofford, D. L. (2002) PAUP*: Phylogenetic analysis using parsimony (*and other methods) (Sunderland, MA: Sinauer Associates).

Towbin, M., Staehelin, T., and Gordon, J. (1979) Electro-phoretic transfer of proteins from polyacrylamide gels to nitrocellulose sheets; procedure and some applications. *Proc. Natl. Acad. Sci. USA* **76**, 4350-4354.

Yakovlev, M.S., and Zhukova, G.Y. (1980) Chlorophyll in embryos of angiosperm seeds, a review. *Bot Not.* **133**, 323-336.

Yamasato, A., Nagata, N., Tanaka, R., and Tanaka, A. (2005) The N-terminal domain of chlorophyllide *a* oxygenase confers protein instability in response to chlorophyll *b* accumulation in *Arabidopsis*. *Plant Cell* **17**, 1585-1597.

Yuan, M., Zhang, D.W., Zhang, Z.W., Chen, Y.E., Yuan, S., Guo, Y.R., and Lin, H.H. (2012) Assembly of NADPH: protochlorophyllide oxidoreductase complex is needed for effective greening of barley seedlings. *J. Plant Physiol.* **169**(13), 1311-1316.

SI Figures

```
  1   MEAALAACAL PSLRILNTKP RFRCSFSNPS LPISPNSLIT RKSSRFTTAV
          *
 51   SSPPSSSAAT STNSPPEPEA LFEPGSDKFD WYANWYPVMP ICDLDKKVPH
101   GKKVMGIDLV VWWDRNEKQW KVMDDTCPHR LAPLSDGRID QWGRLQCVYH
151   GWCFNGSGDC KLIPQAPPDG PPVHTFKQAC VAVYPSTVQH EIIWFWPNSD
201   PKYKNIIETN KPPYIPELED PSFTKLMGNR DIPYGYDVLV ENLMDPAHVP
251   YAHYGLMRFP KPKEKIDREG GKPLEINVKK LDNKGFFSKQ EWGYSNFIAP
301   CVYRSSTDPL PEQEHEYPAP AASDKAALSK RRLSLIFICI PVSPGRSRLI
351   WTFPRNFGVF IDKIVPRWVF HIGQNTILDS DLHLLHVEER KILERGPENW
401   QKACFIPTKS DANVVTFRRW FNKYSEARVD WRGKFDPFLL PPTPPREQLF
                                  ____________________
451   DRYWSHVENC SSCKKAHKYL NALEVILQIA SVAMIGVMAV LKQTTMSNVA
      _________
501   RIAVLVAAVL SFAASKWLSH FIYKTFHYHD YNHAVV
```

Fig. S1. Predicted amino acid sequence of the At4g25650 gene product. The Rieske centre binding motif ($CxHx_{16-17}CxH$) and mononuclear iron binding ($Nx_2Dx_{3-4}Hx$) motif are highlighted in yellow and green colour, respectively. The asterisk marks the potential cleavage site for a chloroplast transit peptide. Predicted *trans*-membrane domains are overlined.

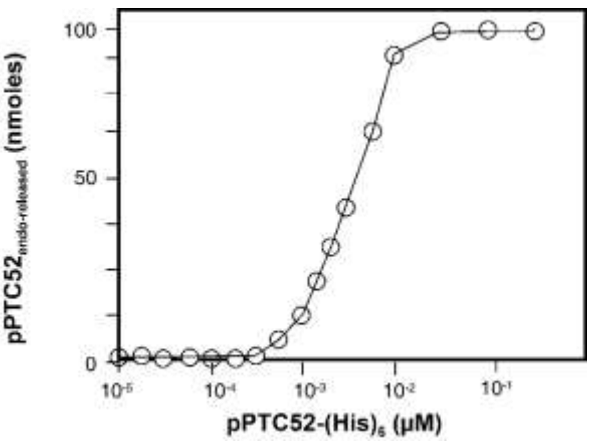

Fig. S2. Production of AtPTC52-$(His)_6$-containing complexes in chloroplasts.
Shown is a dose-response curve of release of the endogenous PTC52 protein from pre-existing PTC complexes *versus* the concentration of added AtPTC52-$(His)_6$. After incubation at the indicated concentrations of AtPTC52-$(His)_6$, the amounts of newly formed and remaining endogenous complexes were determined by non-denaturing PAGE (1) and Western blotting using antisera against PTC52 and $(His)_6$.

1. Reinbothe, S., Krauspe, R., Parthier, B. (1990) Partial purification and characterization of mRNAs for chloroplast and cytoplasmic aminoacyl-tRNA synthetases from *Euglena gracilis*. *J. Plant Physiol.* **137**, 81-87.

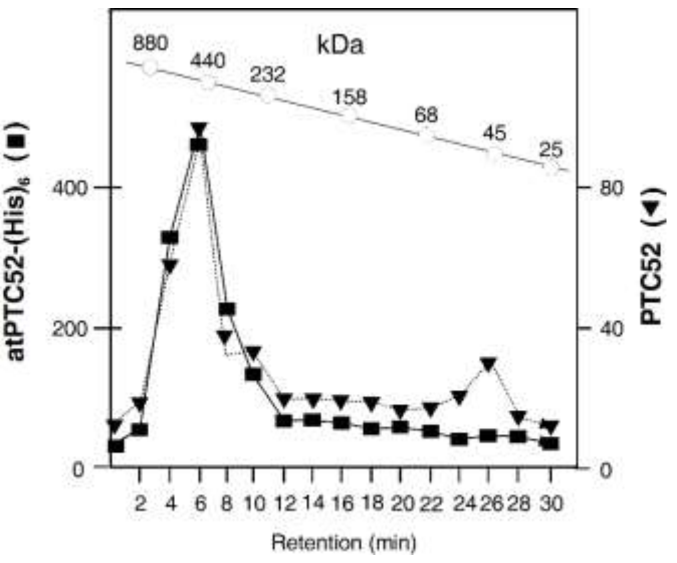

Fig. S3. Size exclusion chromatography to determine the size of PTC52-containing complexes. AtPTC52-(His)$_6$-containing complexes were established with 2.5 nmoles of AtpPTC52-(His)$_6$ and purified by Ni-NTA chromatography from solubilized OM-IM junction complexes. Then, the proteins and complexes bound to AtPTC52-(His)$_6$ were subjected to size exclusion chromatography on a Superose 6 column (1). PTC52-containing complexes were detected by SDS-PAGE and Western blotting using (His)$_6$- specific (solid line) or PTC52-specific antisera (dotted line). Migration of molecular mass standards is indicated.

1. Schemenewitz, A., Pollmann, S., Reinbothe, C., Reinbothe, S. (2007) A substrate-independent, 14:3:3 protein-mediated plastid import pathway of NADPH:protochlorophyllide oxidoreductase (POR) A. *Proc. Natl. Acad. Sci. USA* **104**, 8538-8543.

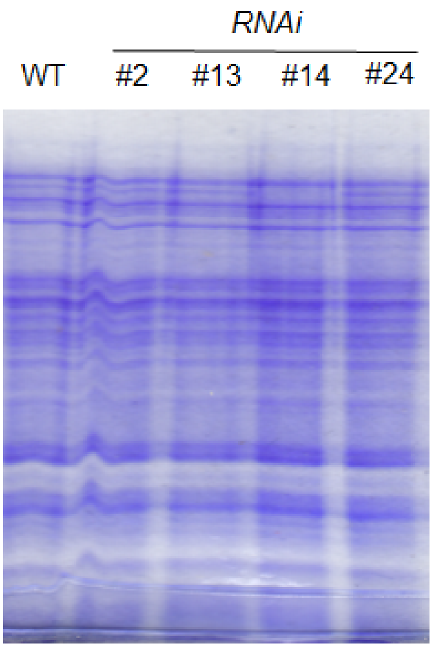

Fig. S4. Overall pattern of Coomassie-stained proteins in 4.5 d-old (DEX)-induced RNAi lines, as compared to the wild-type (WT).

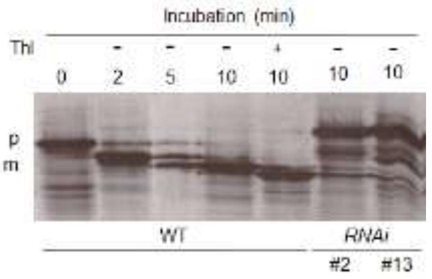

Fig. S5. In vitro import of pPORA into isolated plastids from RNAi lines #2 and #13, as compared to isolated plastids from the wild-type (WT). Precursor (p) and mature (m) protein bands are indicated. Thl stands for thermolysin used to degrade unimported pPORA at the end of the incubation.

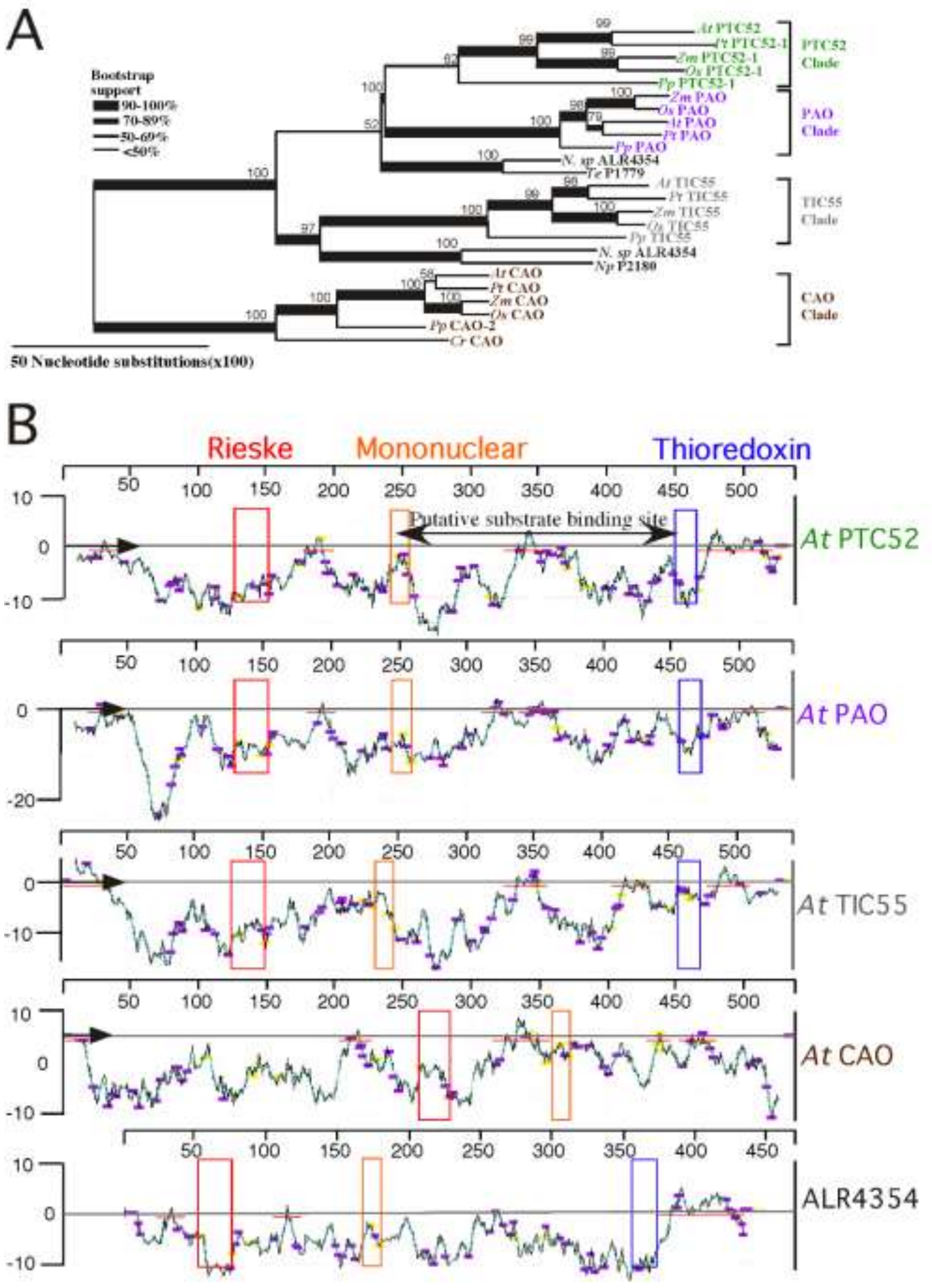

Fig. S6. Evolutionary relationships among PTC52 and related land plant and cyanobacterial non heme oxygenases.

(A) Phylogenetic tree of 25 non-heme oxygenases estimated using maximal parsimony. Proteins are named according to their abbreviated species name followed by the gene name *(Arabidopsis thaliana, At; Chlamydomonas reinhardtii, Cr; Nostoc punctiforme, Np; Nostoc species PCC2170, PCC; Physcomitrella patens, Pp; Populus trichocarpa, Pt; Trichodesmium erythraeum, Te; Oryza sativa, Os; Zea mays, Zm).* A multiple sequence alignment of PTC52-related proteins (see Fig. 1B and Figs S6 and S7) was determined using Clustal W (default parameters). Branch lengths are proportional to the expected number of nucleotide substitutions. The reliability of each bifurcation was estimated using bootstrap analysis (percentage values over 50% are shown next to nodes), and the support for each of

the branches is indicated by line thickness. The tree is drawn using the chlorophyll oxygenase CAO clade as a monophyletic ingroup.

(B) Comparison of hydropathicity plots between *A. thaliana* PTC52-related non-heme oxygenases and *Nostoc* species PCC7120 ALR4354. Hydropathy plots were determined using an experiment-based Wimley-White whole-residue hydrophobicity octanol-interface scale (1) and the Membrane Protein Explorer (MPEx) tool (http://blanco.biomol.uci.edu/mpex/). The hydropathy profile is shown in black with a superimposed version in green. Red bars indicate possible *trans*-membrane or membrane associated regions. Aromatic residues (F, W, and Y) are highlighted in purple and H residues in yellow.

1. White, W.C., and Wimley, S.H. (1999) Membrane protein folding and stability: physical principles. Annu. Rev. Biophys. Biomol. **28**, 319-365.

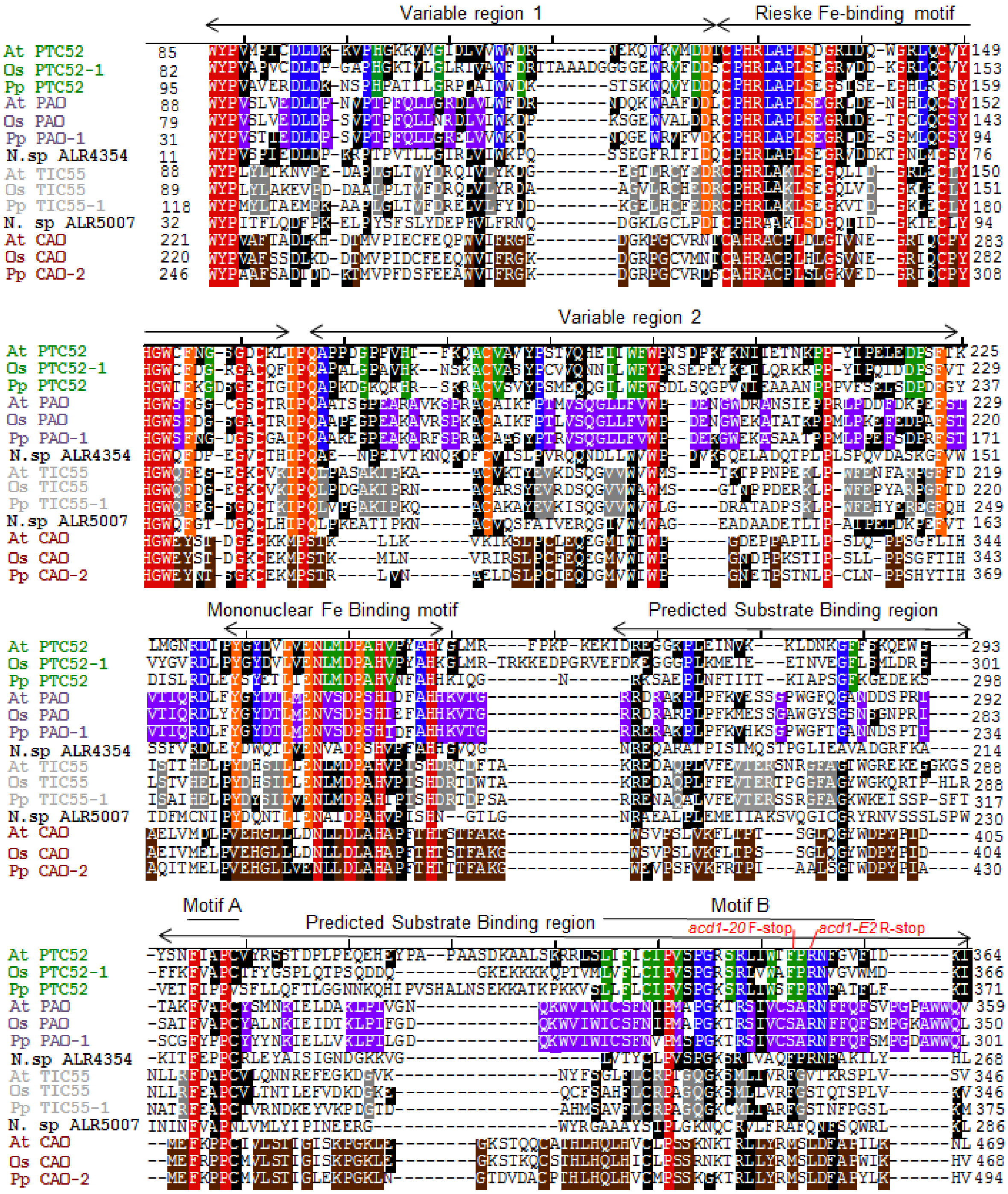

Variable region 1
Rieske Fe-binding motif
Variable region 2
Mononuclear Fe Binding motif
Predicted Substrate Binding region
Motif A
Predicted Substrate Binding region
Motif B
acd1-20 F-stop
acd1-E2 R-stop

Fig. S 7 (continued)

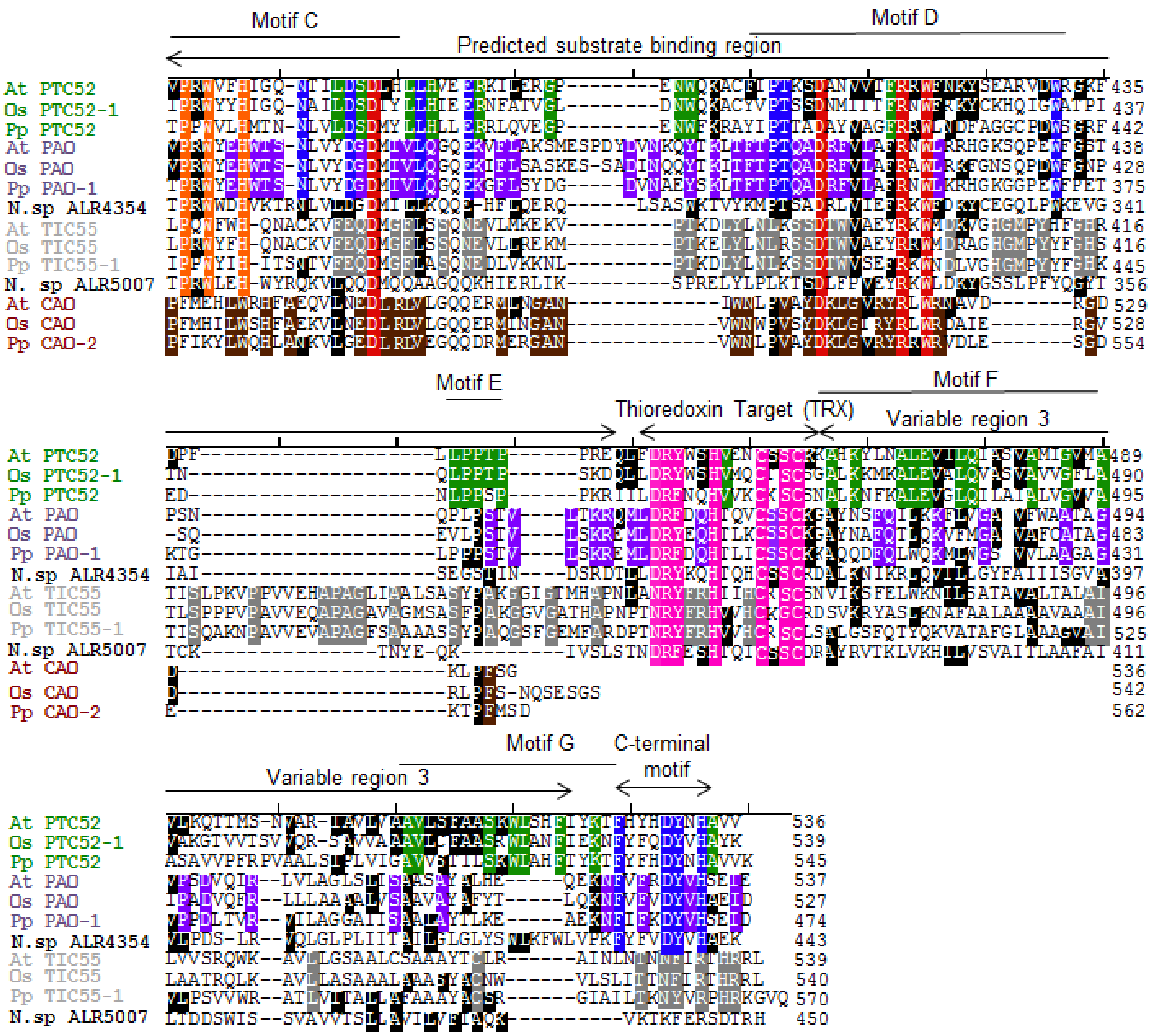

Fig. S7. Amino acid residues conserved in different PTC52 related proteins from land plants.

Multiple sequence alignment of PTC52, PAO, TIC55 and CAO proteins from land plants (*Arabidopsis thaliana* At, *Populus trichocarpa* Pt, *Oryza sativa* Os, *Zea mays* Zm, and *Physcomitrella patens* Pp). Sequences from two related dioxygenases from *Nostoc* species PCC 7120 are also included. Sequences are aligned from the first residue conserved in all proteins and 14 sequences are shown from an original alignment of 25 proteins including 5 land plants and three photosynthetic bacteria. Residues conserved amongst all proteins are shown in red, amongst the PTC52, PAO, and TIC55 subclades in orange, amongst the PTC52 and PAO subclades in blue. Residues conserved uniquely within the PTC52 clade are shown in green, the PAO clade in purple, the TIC55 clade in grey and the CAO clade in brown. Conserved residues in the thioredoxin target motif which is only present in the PTC52, PAO, and TIC55 families, are highlighted in pink. Motifs of known and unknown functions are delineated by horizontal lines.

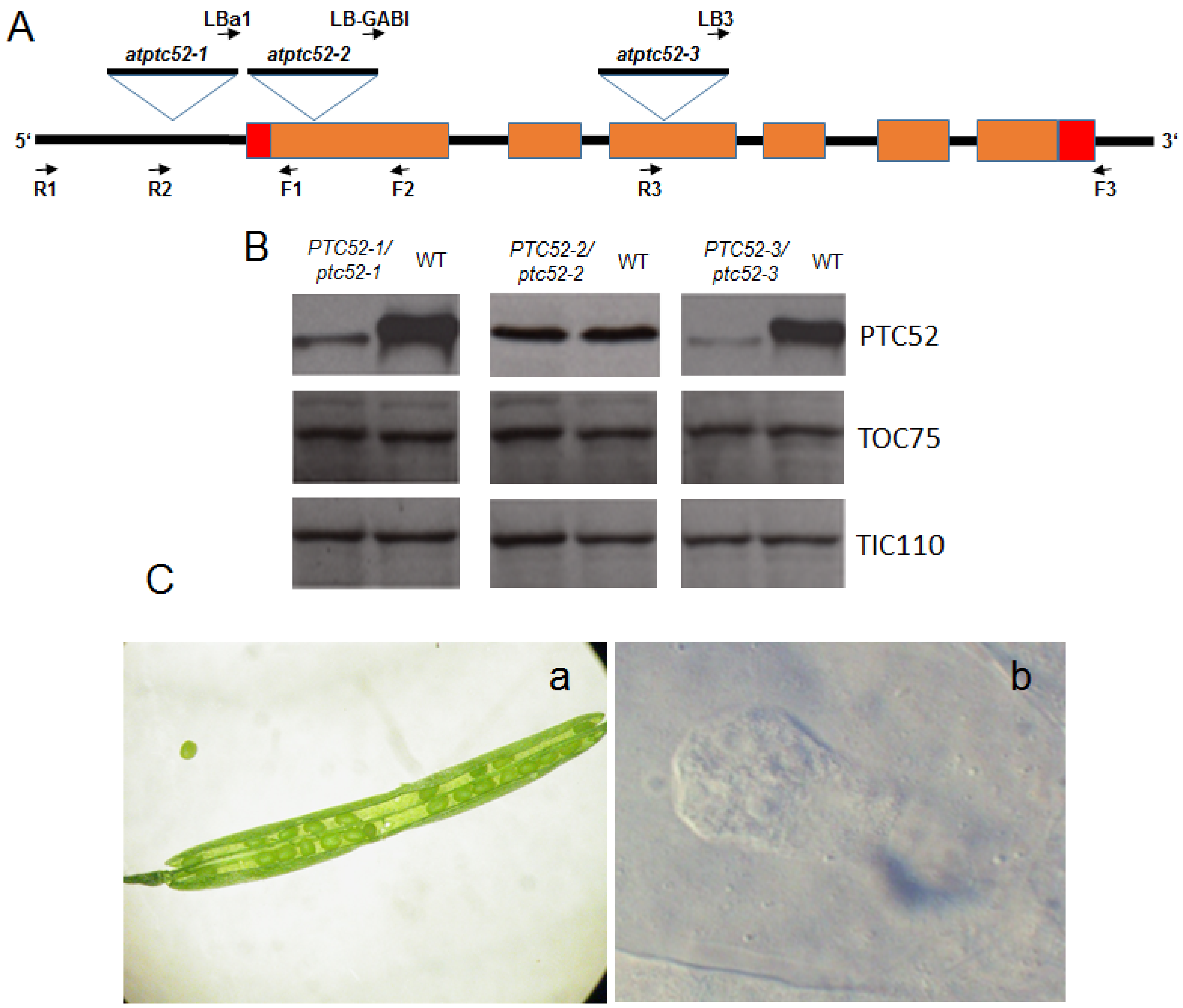

Fig. S8. Structure of the *PTC52* gene, location of mutations and identification of *AtPTC52-1/Atptc52-1*. **(A)** Structure of *AtPTC52* and predicted mutation sites (Salk_011945 [*Atptc52-1* allele]; GK-043G03 [*Atptc52-2* allele] and Garlic_148_H05.b.1a.Lb3Fa; SAIL_148.HC5 [*Atptc52-2* allele). 5'- and 3'-untranslated regions are in red, exons and introns in ochre and black, respectively. The 4.5 kb T-DNA insertions (not drawn to scale) are shown as black bars. Initiation sites for primers used for PCR identification of *Atptc52* alleles are marked.

(B) Western blot of PTC52, TOC75 and TIC110 in chloroplasts of 4 weeks-old wild-type (WT) and *AtPTC52-1/Atptc52-1, AtPTC52-2/Atptc52-2* and *AtPTC52-3/Atptc52-3* plants.

(C) Examination of a typical silique of self-crossed *AtPTC52-1/Atptc52-1* plants by light microscopy (panel a). The empty areas sre due to aborted embros. Arrest of embryo development was revealed by Nomarski optics (panel b).

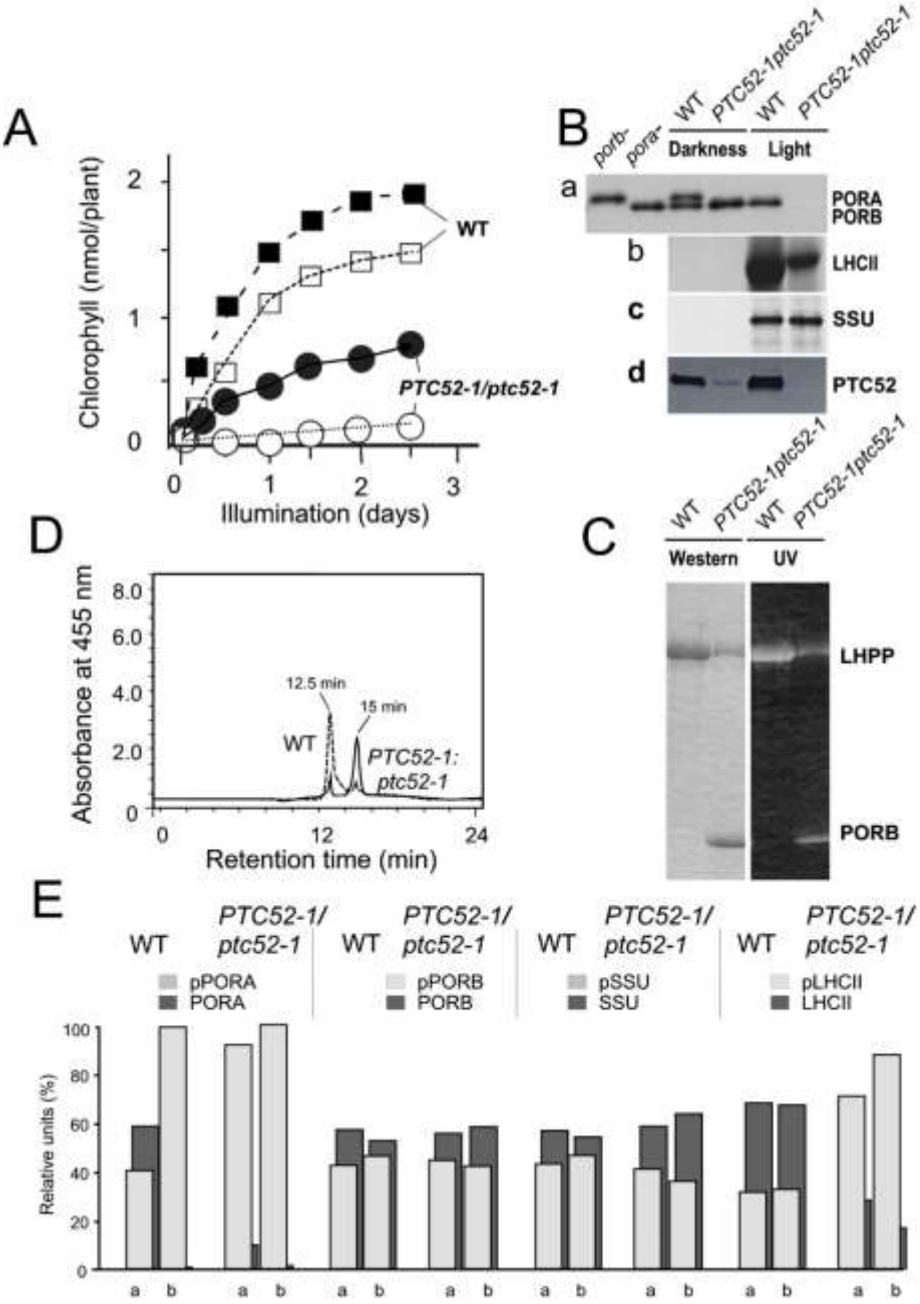

Fig. S9. Chlorophyll and protein accumulation kinetics of *AtPTC52-1/Atptc52-1* seedlings.

(A) Chlorophyll accumulation kinetics determined for 5 d-old, dark-grown *AtPTC52-1/Atptc52-1* (filled and open circles) and wild-type seedlings (filled and open squares) that had been exposed to white light of 5 µE m^{-2} sec^{-1} (filled circles and squares) or 125 µE m^{-2} sec^{-1} (open circles and squares) for the indicated periods.

(B) POR abundance in etioplasts and etiochloroplasts of 5 d-old dark-grown *AtPTC52-1/Atptc52-1* and wild-type seedlings (Darkness) as well as 4 d-old mutant and wild-type seedlings that had been exposed to white light of 125 µE m^{-2} sec^{-1} for 24 h (Light)(panel a). For comparison, etioplast protein extracts were prepared from *A. thaliana porb*- and *pora*-knock-out plants and analyzed in parallel. As references, replicate Western blots were probed with antisera against LHCII (panel b), SSU (panel c) or PTC52 (panel d).

(C) Detection of light-harvesting POR:Pchlide (LHPP) as well as PORB-pigment complexes in etioplasts of dark-grown *AtPTC52-1/Atptc52-1* and wild-type seedlings by Western blotting using POR antiserum (left panel) and pigment fluorescence under UV light (right panel).
(D) HPLC pattern of pigments present in dark-grown *AtPTC52-1/Atptc52-1* (solid line) and wild-type (dashed line) seedlings.
(E) *In vitro*-import of ^{35}S-pPORA, ^{35}S-pPORB, ^{35}S-pSSU and ^{35}S-pLHCII into chloroplasts isolated from 4 weeks-old light-grown *AtPTC52-1/Atptc52-1* and wild-type seedlings. To study the substrate-dependent import of pPORA, an aliquot of the chloroplast suspension was pretreated with a 0.5 mM aqueous solution of 5-aminolevulinic acid to give rise to intraplastidic Pchlide synthesis (lane a in each data set). Mock incubations contained phosphate buffer (lane b). Following import, the plastids were sedimented and aliquots were treated with or without thermolysin (Thl) to allow precursor and mature protein levels to be distinguished by SDS-PAGE and autoradiography or liquid scintillation counting. Light grey and dark-grey columns define precursor and mature protein levels, respectively. Percentages refer to the sum of precursor and mature protein levels in the assays, set as 100.

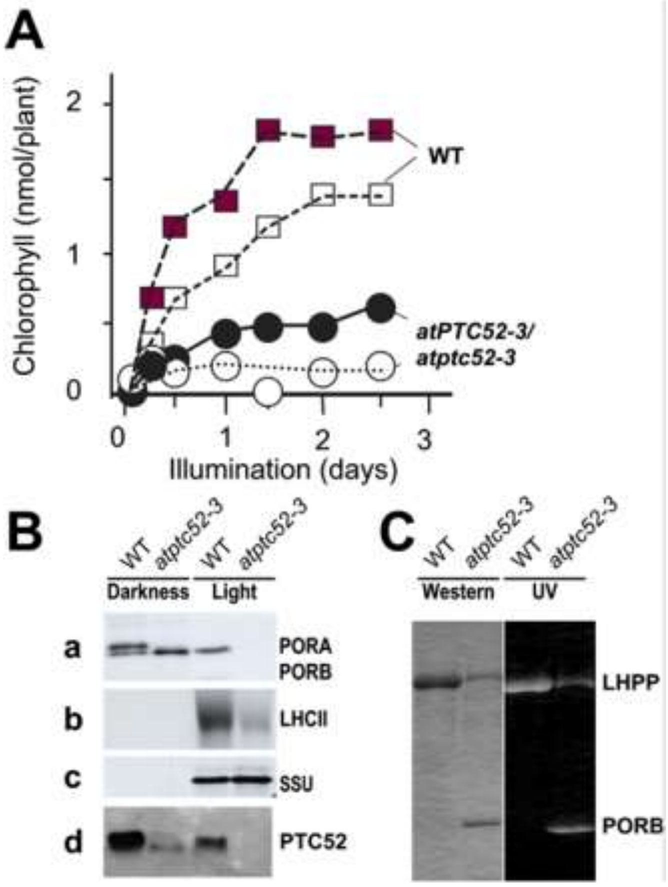

Fig. S10. Greening defect of *AtPTC52-3/Atptc52-3* plants.

(A) Chlorophyll accumulation kinetics determined for 5 d-old, dark-grown *AtPTC52-3/Atptc52-3 (*filled and open circles) and wild-type seedlings (filled and open squares) that had been exposed to white light of 5 µE m-2 sec-1 (filled circles and squares) or 125 µE m-2 sec-1 (open circles and quares) for the indicated periods.

(B) POR abundance in etioplasts and etiochloroplasts of 5 d-old dark-grown *AtPTC52-3/Atptc52-3* and wild-type seedlings (Darkness) as well as 4 d-old mutant and wild-type seedlings that had been exposed to white light of 125 µE m^{-2} sec^{-1} for 24 h (Light)(panel a). For comparison, replicate Western blots were probed with antisera against LHCII (panel b), SSU (panel c) or PTC52 (panel d).

(C) Detection of light-harvesting POR:Pchlide (LHPP) as well as PORB-pigment complexes in etioplasts of dark-grown *AtPTC52-3/Atptc52-3* and wild-type seedlings by Western blotting using POR antiserum (left panel) and pigment fluorescence under UV light (right panel).

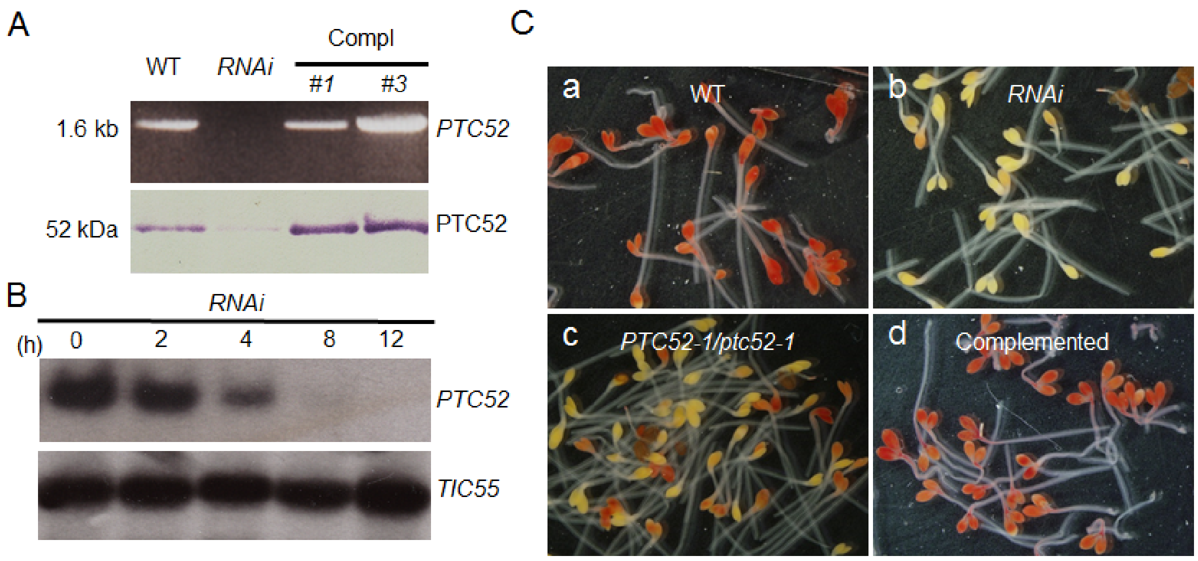

Fig. S11. Identification and characterization of genetically complemented *AtPTC52-1/Atptc52-1* plants and *RNAi* plants lacking *AtPTC52* transcript and AtPTC52 protein.

(A) Expression analysis of *AtPTC52* transcript and AtPTC52 protein in wild-type (WT), *RNAi* and genetically complemented *AtPTC52-1/Atptc52-1* seedlings, as revealed by RT-PCR (upper panel) and Western blotting using specific antibodies (lower panel), respectively.

(B) Time course of *AtPTC52* transcript abundance in 4 d-old etiolated seedlings treated with dexamethasone for the indicated time periods, analysed by Northern blotting (upper panel). The lower panel shows *AtTIC55* transcript levels for comparison.

(C) Viability of etiolated wild-type (a), *RNAi* (b), *AtPTC52-1/Atptc52-1* (c) and genetically complemented *AtPTC52-1/Atptc52-1* seedlings (d) after a 30-min light exposure, as assessed by tetrazolium staining. While viable seedlings retain the red dye, dead seedlings do not and look whitish-yellow.

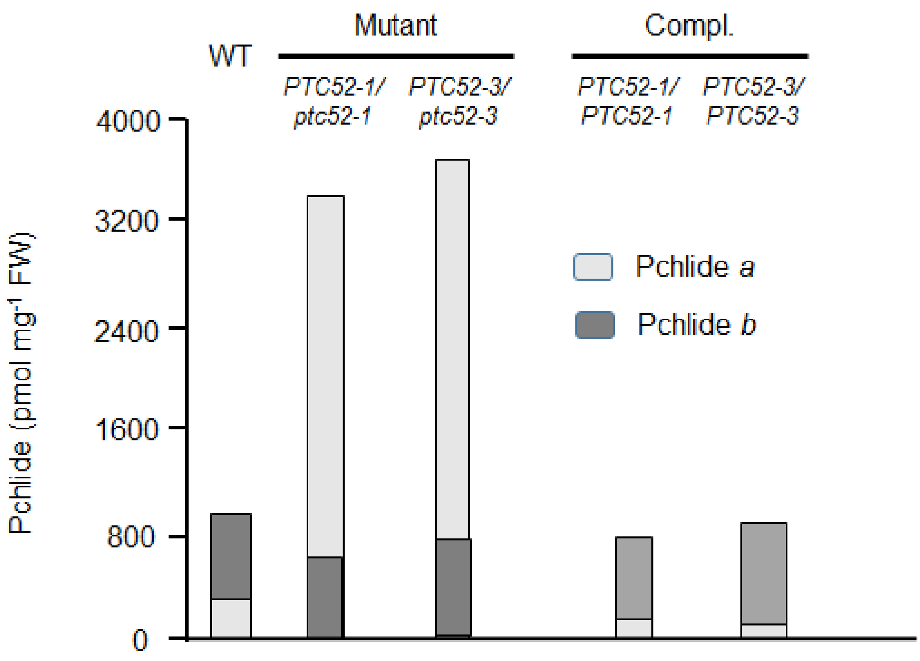

Fig. S12. Restoration of wild-type levels of Pchlide *a* and Pchlide *b* in genetically complemented *AtPTC52-1/Atptc52-1* and *AtPTC52-1/Atptc52-1* plants. Seedlings were grown for 4.5 d in karness to provoke etiolation and pigments extraction and quantification made by HPLC. Note the perturbed Pchlide *a*-to-*b* ration in the parental *AtPTC52-1/Atptc52-1* and *AtPTC52-3/Atptc52-3* lines, as compared to the wild-type, and that this is restored by genetic transformation.

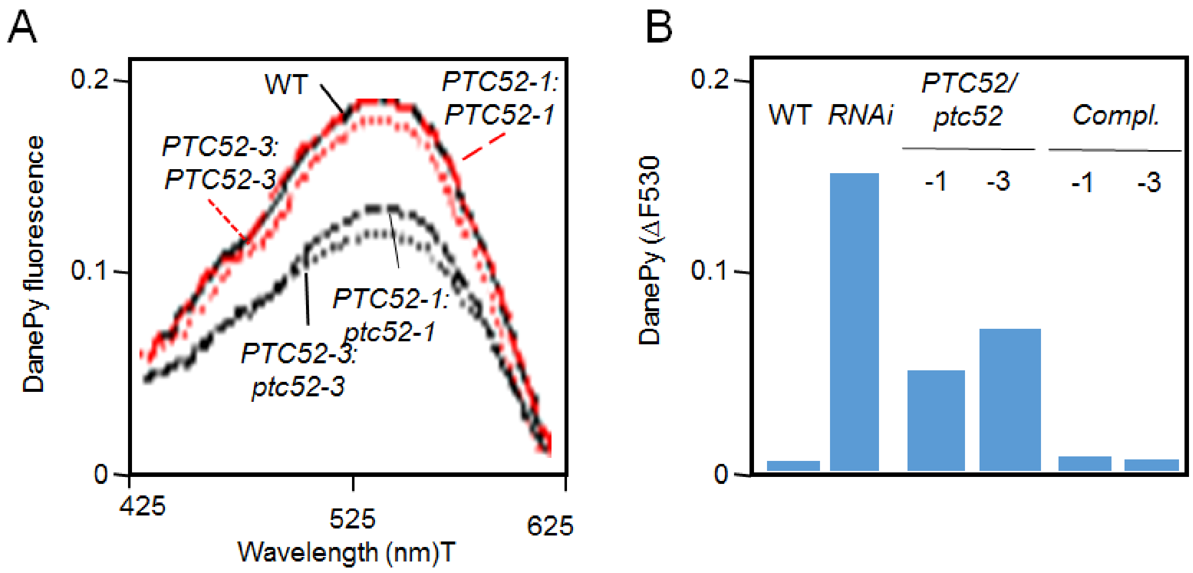

Fig. S13. Light-triggered singlet oxygen production in etiolated wild-type (WT), *AtPTC52-1/Atptc52-1* mutant, genetically complemented *AtPTC52-1/Atptc52-1*, and RNAi seedlings.

(A) Fluorescence analysis of DanePy used as singlet oxygen quencher in 4.5 d-old etiolated seedlings after a 30-min light exposure. The different curves show DanePy fluorescence spectrta for wild-type (WT) seedlings (black line), *AtPTC52-1/Atptc52-1* mutant seedlings (black hatched line), *AtPTC52-3/Atptc52-3* mutant seedlings (black dotted line), genetically complemented *AtPTC52-1/Atptc52-1* mutant seedlings (red hatched line) and genetically complemented *AtPTC52-3/Atptc52-3* mutant seedlings (red dotted line).

(B), as **(A),** but showing a quantitation of DanePy quenching and thus singlet oxygen production for wild-type (WT), *RNAi*, *AtPTC52-1/Atptc52-1* and *AtPTC52-1/Atptc52-1* mutant and respective genetically complemented lines. Note the inverse correlation between the amount of singlet oxygen and seedling viability in the different mutants and genetically complemented lines (cf. Fig. 4).

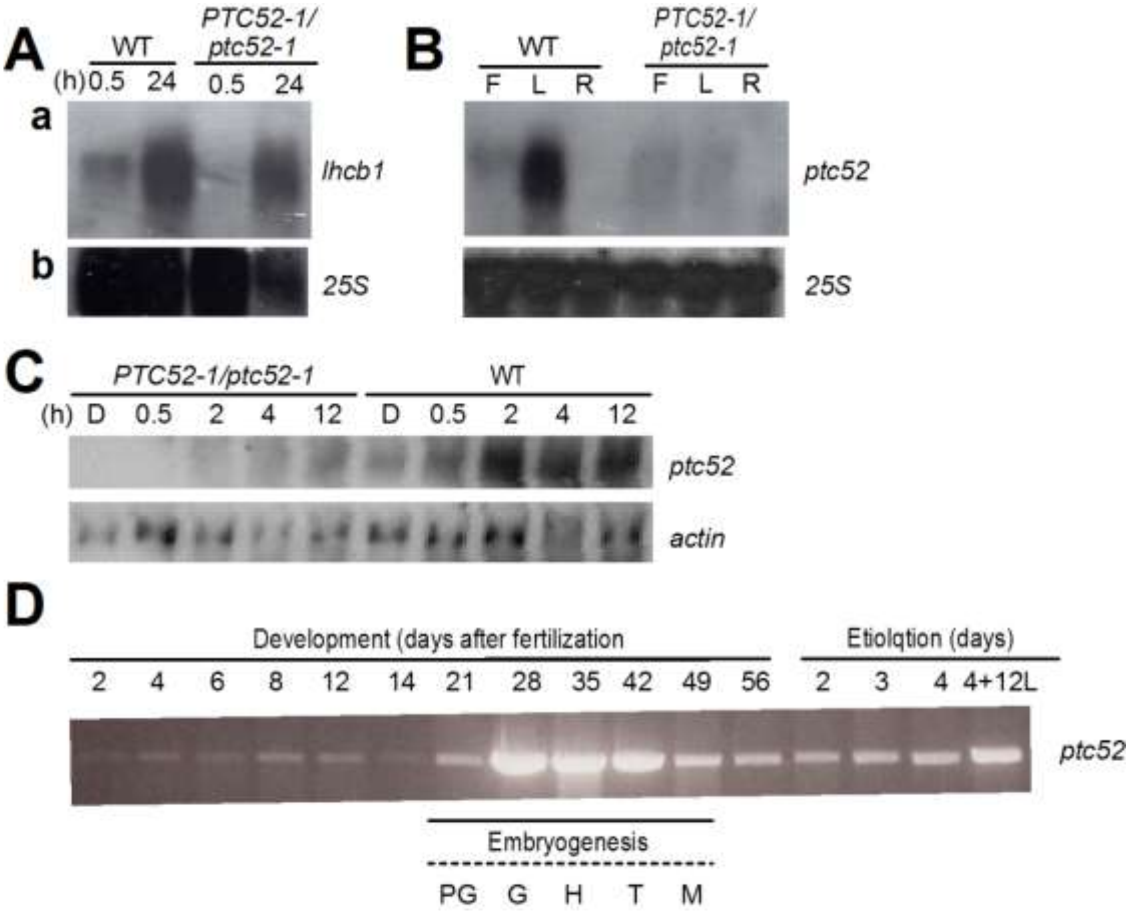

Fig. S14. Expression of *PTC52* over plant development.

(A) Wild-type (WT) and *AtPTC52-1/Atptc52-1* seeds were grown in the dark for 4 days and subsequently exposed to white light for the indicated periods. mRNA was isolated and separated on agarose gels. After blotting, filter-bound RNAs were hybridized with a *LHCB1* probe. As control, a replicate filter was hybridized with a 25S rRNA probe.

(B), as in (A), but showing the expression of *PTC52* transcripts and 25S rRNA in flowers (F), leaves (L) and roots (R) of 6 weeks-old *AtPTC52-1/Atptc52-1* and wild-type plants.

(C), as in (A), but depicting the expression profile of *PTC52* and *ACTIN* transcripts amongst total RNA during greening of 5-days-old *AtPTC52-1/Atptc52-1* and wild-type seedlings.

(D) RT-PCR analysis to follow the expression of *PTC52* in flowers and siliques after fertilization, giving rise to proglobular (PG), globular (G), heart-staged (H), torpedo-staged (T) and mature (M) embryos, as well as during seedling growth in darkness (etiolation) and subsequent greening triggered by a 12h shift to white light of 125 µE m^{-2} sec^{-1}.

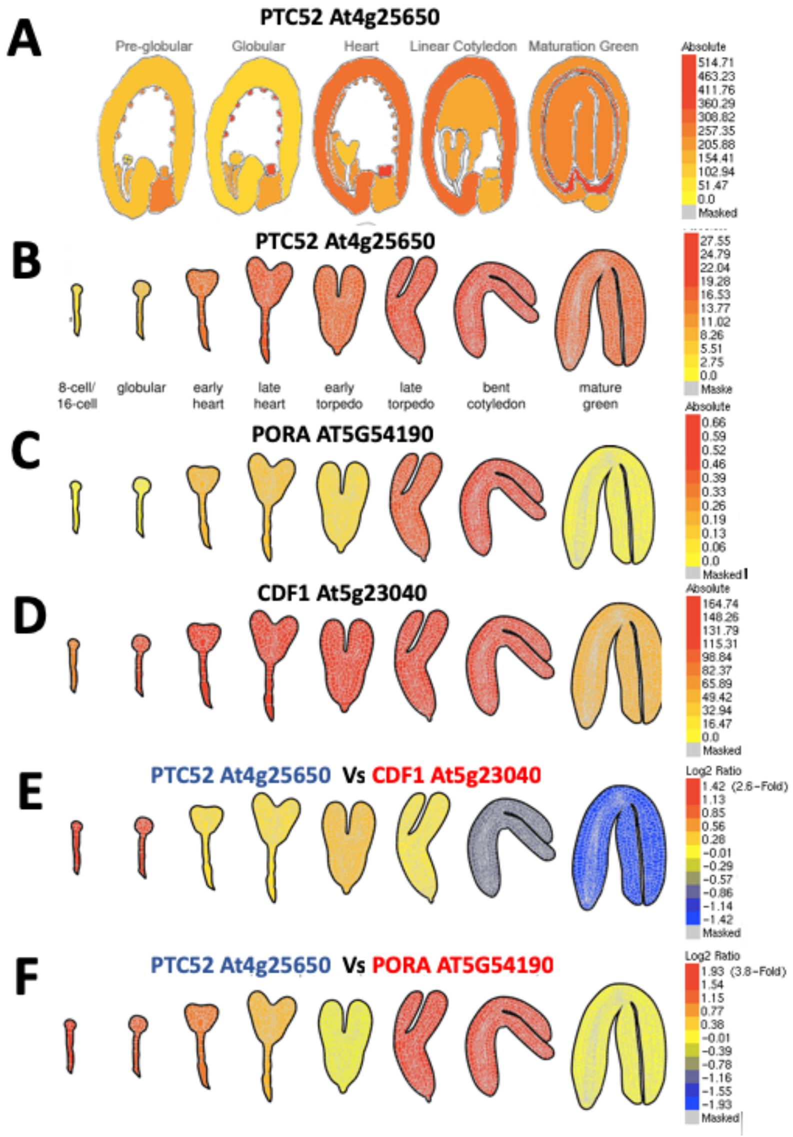

Supp. Fig. 15: Spatial, temporal and comparative expression profiles of PTC52, PORA and CDF1 in developing *A. thaliana* seeds and embryos. All data were retrieved from The Bio-Analytic Resource for Biology (http://www.bar.utoronto.ca/).

(A) Electronic fluorescent pictographs (eFPs) of PTC52 (At4g25650) in *Ws* Arabidopsis seeds. The indicated tissues at various developmental stages were excised by laser-capture microdissection and hybridized to ATH1 GeneChip as described by Le et al. 2010).

(B) eFP of PTC52 (At4g25650) in developing Arabidopsis embryos. Plants were grown in a light grown chamber at 20-22°C with a 16-h light/8-h dark cycle. RNA was extracted and subjected to RNA-Seq analysis as described by Hofmann *et al.,* 2019. Expression is shown on an absolute scale.

(C, D) eFP as in (B) except showing data for PORA (At5g54190) and CDF1 (At5g23040)

(E, F) Comparative expression eFP of PTC52 (At4g25650) versus CDF1 (At5g23040) and of PTC52 (At4g25650) versus PORA (At5g54190) respectively. Expression is shown as a Log_2 ratio. Data from Hofmann et al., 2019.

Table S1. Conserved residues in predicted substrate binding domains of PTC52 related plant proteins [1]

Motif	PTC52* PAO* TIC55* CAO	PTC52 Alone	PAO alone	Motif	PTC52* PAO* TIC55* CAO	PTC52 alone	PAO Alone	PTC52* PAO* TIC55* CAO	PTC52 alone	PAO alone
	E241					F337	S326	L385		
E/D,Nx2Hx4H	N242						F327		H386	Q381
			V247	C/N,xP	C/N 339		N328	E389		
			S248			I340			R390	K385
	D245*				P341					F387
	P246*					V342	M331			L388
		A247	S251		P344				G396	
	H248				G345					D398
		V249	I253			S347				N400
	A252				R348				N399	
	H253						S338		W400	Y403
			H258			W351	V340			K405
			K259				C341			T407
			V260				S342			F408
			T261			P354	A344			T409
			G262		R355			P407		
			R266		N356			T408		
	P273						F347			Q412
			S277				Q348			A413
			G278				F349	D411		
			W280				P352			R415
	G285*						G353			F416
		F286					A355			L418
			N286				W356		F417	
			I292				W357	R418		
FxA/P,P,C/V	F297						Q358	W420		
	A/P 299				P366*					L424
	P300				W368*			W431		
	C/V 301						E365		L440	
			Y301		H371*				P441	
			K305				W367	P442		
			K311				T368	T/S 443		
			L312				S369			V448
			P313		N375					L449
			I314				Y373			K451
			Q318		D379					R452
			K319			S380	G375			M454

W320	D381	L455
L335 I324	I378	
C325	V379	

Table S2 Pigment accumulation in heterozygous *AtPTC52-1/Atptc52-1* plants

	Pigment (pmol per g fresh weight)	
	WT	*PTC52-1/ptc52-1*
**Darkness (4.5d D)**		
Pchlide *a*	250±35	3250±275
Pchlide *b*	1050±185	375±70

Illuminated (4d D + 12h WL)

	Low light		Medium light		High light	
	WT	*PTC52-1 ptc52-1*	WT	*PTC52-1 ptc52-1*	WT	*PTC52-1 ptc52-1*
Pchlide *a*	225±15	2750±225	270±30	2250±250	*245*±30	2*75*±100
Pchlide *b*	550±35	225±20	275±75	350±80	150±35	325±5
Chlide *a*	1000±70	500±100	2500±200	25±10	3000±75	n.d.
Chlide *b*	550±125	50±15	850±125	25±5	1250±25	n.d.

Seedlings were grown for 5 d in darkness and pigments were extracted with 100 % acetone containing 0.1 % diethylpyro-carbonate (DEPC) and subjected to HPLC as described previously (Reinbothe et al., 2003a,2003b). In a parallel experiment, seedlings were germinated for 4 d in the dark and transferred to white light of 5 µE m^{-2} sec^{-1} (low light), 125 µE m^{-2} sec^{-1} (medium light) or 250 µE m^{-2} sec^{-1} (high light)for 24 h. Identification and quantification of the HPLC-resolved pigments was made by absorbance measurements using a photodiode array detector and known standards. Mean values refer to three independent experiments.
